# APNet, an explainable sparse deep learning model to discover differentially active drivers of severe COVID-19

**DOI:** 10.1093/bioinformatics/btaf063

**Published:** 2025-02-08

**Authors:** George I Gavriilidis, Vasileios Vasileiou, Stella Dimitsaki, Georgios Karakatsoulis, Antonis Giannakakis, Georgios A Pavlopoulos, Fotis Psomopoulos

**Affiliations:** Institute of Applied Biosciences, Centre for Research and Technology Hellas, Thessaloniki, GR57001, Greece; Institute of Applied Biosciences, Centre for Research and Technology Hellas, Thessaloniki, GR57001, Greece; Department of Molecular Biology and Genetics, Democritus University of Thrace, Alexandroupolis, GR68100, Greece; Institute of Applied Biosciences, Centre for Research and Technology Hellas, Thessaloniki, GR57001, Greece; Institute of Applied Biosciences, Centre for Research and Technology Hellas, Thessaloniki, GR57001, Greece; Department of Molecular Biology and Genetics, Democritus University of Thrace, Alexandroupolis, GR68100, Greece; University Research Institute of Maternal and Child Health and Precision Medicine, National and Kapodistrian University of Athens, Athens, GR11527, Greece; Institute for Fundamental Biomedical Research, BSRC “Alexander Fleming”, Vari, GR16672, Greece; Center of New Biotechnologies & Precision Medicine, Department of Medicine, School of Health Sciences, National and Kapodistrian University of Athens, Athens, GR11528, Greece; Institute of Applied Biosciences, Centre for Research and Technology Hellas, Thessaloniki, GR57001, Greece

## Abstract

**Motivation:**

Computational analyses of bulk and single-cell omics provide translational insights into complex diseases, such as COVID-19, by revealing molecules, cellular phenotypes, and signalling patterns that contribute to unfavourable clinical outcomes. Current in silico approaches dovetail differential abundance, biostatistics, and machine learning, but often overlook nonlinear proteomic dynamics, like post-translational modifications, and provide limited biological interpretability beyond feature ranking.

**Results:**

We introduce APNet, a novel computational pipeline that combines differential activity analysis based on SJARACNe co-expression networks with PASNet, a biologically informed sparse deep learning model, to perform explainable predictions for COVID-19 severity. The APNet driver-pathway network ingests SJARACNe co-regulation and classification weights to aid result interpretation and hypothesis generation. APNet outperforms alternative models in patient classification across three COVID-19 proteomic datasets, identifying predictive drivers and pathways, including some confirmed in single-cell omics and highlighting under-explored biomarker circuitries in COVID-19.

**Availability and implementation:**

APNet’s R, Python scripts, and Cytoscape methodologies are available at https://github.com/BiodataAnalysisGroup/APNet.

## Graphical Abstract

**Graphical Abstract:**
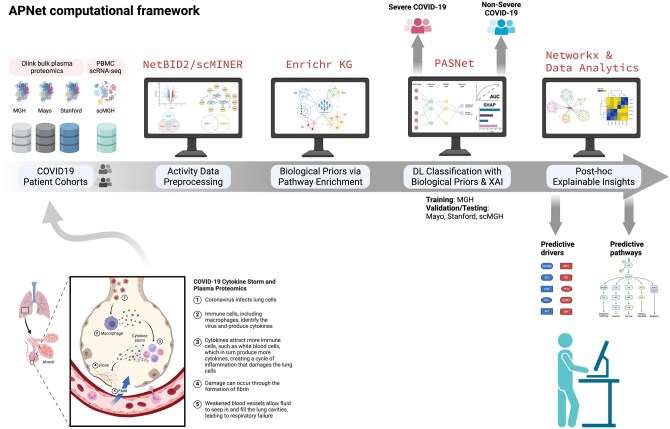

## 1 Introduction

Machine learning (ML) has significantly advanced biomedical research by computationally deconvoluting high-throughput omic datasets to yield insights into potentially novel biomarkers, signalling pathways, druggable targets, and patient stratification schemas. Deep learning (DL), a more intricate form of ML inspired by the neuronal operations of the human brain, has emerged as an even more transformative computational approach in omic research. It can autonomously learn hierarchical representations from raw data, making it essential for identifying complex dependencies within datasets, even in the presence of noise and high-dimensional data. However, DL models often operate as ‘black boxes’, lacking transparency and understandability for humans. This opacity presents a challenge, particularly in the biomedical sciences, where justifying AI-based decisions is critical. Consequently, research in explainable AI (XAI) is growing, aiming to develop AI models that are more understandable to humans while maintaining or improving their performance (Santorsola and Lescai 2023).

ML and DL computational models on multi-omics with variable degrees of XAI have been pivotal in the fight against SARS-CoV-2 infections that have caused the 2019 global COVID-19 pandemic. This pestilential threat has resulted in over 700 million infections and 7 million deaths worldwide. Although currently, COVID-19 is not posing an immediate threat to public health systems on a pandemic level, the intricate immunopathology of this infectious disease, the emergence of ‘long-COVID-19 syndromes’ post-infection, the need to develop novel antivirals, and the prospect of facing similar threats in the near future continue to drive research endeavours in this field (Jamison *et al.* 2022, Williams *et al.* 2023, Narayanan *et al.* 2024).

Plasma proteomics encompasses a broad spectrum of proteins from the peripheral blood, including tissue markers, immunoglobulins, transcription factors, kinases, metabolites, and secreted factors, and is usually a focal point in COVID-19 studies (Zhong *et al.* 2021, Eldjarn *et al.* 2023). This is particularly relevant in severe COVID-19, which besets many patients infected with SARS-CoV-2, and involves an inflammatory ‘cytokine storm’, Acute Respiratory Distress Syndrome (ARDS), PANoptosis-induced cell death, and multiorgan failure (Diamond and Kanneganti 2022, Babačić *et al.* 2023). Plasma proteomics is often analysed together with single-cell omic approaches, like scRNA-seq, to trace critical circulating proteins’ otherwise-obscure cellular origin to specific cell groups of potential translational interest (Feyaerts *et al.* 2022).

Many studies have measured plasma proteomics using Olink Proximity Extension Assay (PEA) in COVID-19 research due to this technology’s specificity, scalability, and multiplexing benefits (Wik *et al.* 2021). In our recent work, we assessed pertinent ML models applied to these high-dimensional datasets, such as Random Forest, Gradient Boosted Decision Trees, XGBoost, Extra Trees, Logistic Regression, Lasso Logistic Regression, Support Vector Machines (SVM), and DL (e.g. AutoGluon-Tabular). Some models exhibited eXplainable AI (XAI) features by deploying Shapley additive explanation (SHAP) values, the minimal-optimal variables method, or a random forest explainer. In the same work, we integrated an explainable computational pipeline to benchmark a wide assortment of ML tools on predicting COVID-19 severity from Olink plasma proteomics, which revealed Multi-Layer Perceptron (MLP) as the highest-performing algorithm (Dimitsaki *et al.* 2023).

However, most of these studies can only partially approximate proteomic nonlinear dynamics (e.g. post-translational modifications, protein co-expression networks, complex formation, and subcellular localisation), thus missing signalling proteins that may drive critical COVID-19 pathways. Moreover, the outcomes of these ML/DL studies often require more extensive external validation in large, independent datasets, while their biological explainability is usually restricted to feature ranking (Dimitsaki *et al.* 2023, Paul *et al.* 2023). Furthermore, most ML/DL pipelines in COVID-19 research are complex to repurpose for data integration between plasma proteomics and other omic modalities (e.g. scRNA-seq).

Acknowledging these challenges, in this work, we contextualize a DL framework that addresses data normalisation, data integration, incorporation of biological priors, eXplainable AI (XAI), and network biology. Within this framework, we present a novel biologically informed DL classifier, *Activity PASNet (APNet)*, designed primarily to process Olink plasma proteomics and seamlessly bridge them with other omic modalities. APNet carries out two key tasks:

1. Supervised clustering to distinguish severe from nonsevere COVID-19 cases, and
2. Biological insight generation by creating a protein-pathway bipartite graph enriched with protein/gene regulatory motifs and feature-importance weights derived from the DL component.

APNet harmonizes omic expression values by transforming them into activity matrices based on the SJARACNe algorithm, thereby uncovering the underlying regulatory protein or gene networks (PRN or GRN, respectively) (Dong *et al.* 2023). Furthermore, APNet uses the PASNet architecture as the main biologically informed DL backbone of the entire pipeline for supervised clustering and preliminary biological explainability (Hao *et al.* 2018). APNet also incorporates SHAP values for enhanced interpretability regarding the most predictive molecules from the plasma proteome (Hao *et al.* 2018, Dong *et al.* 2023).

We extensively trained, validated, and tested APNet on activity matrices derived from three distinct Olink plasma proteomic datasets (MGH, Mayo, Stanford) (Filbin *et al.* 2021, Byeon *et al.* 2022, Feyaerts *et al.* 2022) and from two PBMC scRNA-seq datasets (https://www.covid19cellatlas.org/). APNet robustly classified severe COVID-19 cases, pinpointed ground-truth drivers of severity, identified novel hidden drivers of severity, and highlighted bioenergetic perturbations in the liver. Additionally, APNet surpassed other models in benchmarking studies not only in terms of accuracy and robustness but also in the extent to which it uncovered COVID-19 ground truths.

## 2 Materials and methods

### 2.1 APNet overview

APNet is a modular pipeline that aims to facilitate the discovery of novel predictive drivers of severe clinical outcomes and the formulation of mechanistic hypotheses. In this present work, we considered cases experiencing severe and nonsevere COVID-19.

More specifically, APNet constitutes a novel computational framework focused on a biologically informed neural network with enhanced biological explainability for supervised clustering of COVID-19 patients.

APNet entails the following computational ‘building blocks’ (for an extended description of the tools involved, please see Materials and Methods):

1. *NetBID2 and scMINER tools*, which reverse-engineer context-specific Protein Regulatory Networks (PRNs) or Gene Regulatory Networks (GRNs) based on the SJARACNe algorithm (Dong *et al.* 2023). These in-silico tools transform typical expression matrices of omic datasets into ‘activity’ matrices, reflecting the capacity of transcription factors and signal drivers to regulate their target proteins/genes. This biologically inspired form of data preprocessing has revealed, in several leukemias, not only strongly differentially regulated disease drivers but also nuanced and subtle drivers experimentally corroborated yet missed by typical differential expression/abundance analysis (so-called ‘hidden’ drivers).
2. *PASNet (Pathway-associated sparse deep neural network)*, which ingests the activity values (from NetBID2 or scMINER) and uses biological priors [pathway–gene/protein associations from external databases like Enrichr-KG (Evangelista *et al.* 2023)] to perform supervised clustering, prioritizing the most predictive pathways and genes/proteins through assigned learning weights (Hao *et al.* 2018).
3. *SHAP values (SHapley Additive exPlanations)*, which estimate each feature’s influence on the DL output via a linear function that keeps other features constant. The coefficient of this function is the Shapley value. SHAP offers (i) a solid theoretical basis for interpretability, (ii) broad applicability, and (iii) independence from perturbing the model or data.

**Figure 1.**
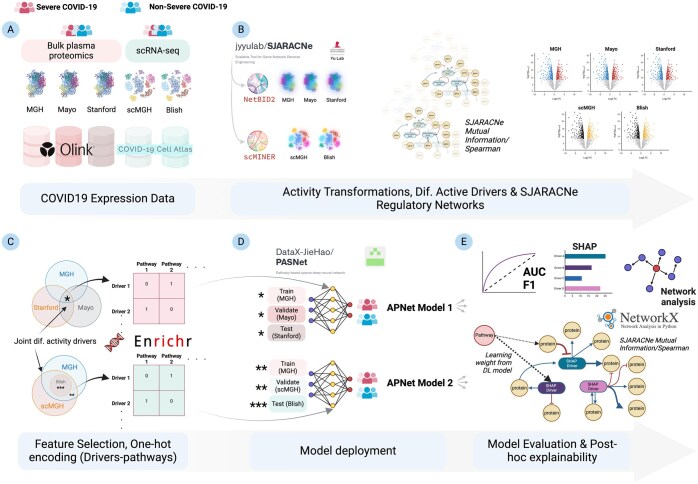
APNet framework as implemented in the herein COVID-19 multi-omic study to discover predictive drivers of severity, and create complex graphs for uncovering non-intuitive connections among drivers and pathways. APNet entails initial pre-processing of COVID-19 bulk plasma proteomics and scRNA-seq (Α) through NetBID2 and scMINER tools (Β) which leads to the retrieval of activity matrices from expression matrices. After SJARACNe regulatory networks and differential activity analysis (Β), key drivers are mapped to pathways through the Enrichr Knowledge Graph (KG) through one-hot mapping (C). Then, activity values of differentially active drivers and the driver-pathway mappings are ingested by sparse neural network models called PASNet which enables supervised clustering of cases in severe and non-severe COVID-19 (D). Ultimately, APNet facilitates the assembly of network models that combine drivers with pathways along with a variety of node weights (e.g., differential activity) and edge weights (e.g., Mutual Information metric, learning weights from PASNet) allowing the leveraging of actionable insights and the in silico testing of biological hypotheses (E). The figure was created with Biorender software.

By combining the PRNs/GRNs from NetBID2/scMINER and the learning weights and SHAP values from PASNet predictions, APNet outputs a protein or gene–pathway bipartite network to aid in formulating mechanistic hypotheses for clinically predictive molecules and signalling motifs. Figure 1 schematically represents the main workflow of APNet.

We structured our classification experiments following DOME recommendations (Walsh *et al.* 2021) for a systematic approach.

### 2.2 Bulk proteomic APNet model

Using NetBID2, we performed independent differential activity analysis on three plasma proteomic datasets (MGH, Mayo, Stanford) to avoid data leakage. Differential activity was calculated within each dataset, and 333 commonly active proteins were identified. One-hot encoding then mapped these proteins to pathways from the Enrichr Knowledge Graph (KG), yielding a matrix of 250 proteins and 210 pathways as prior biological knowledge. The PASNet DL model received the MGH activity matrix as input plus the protein–pathway map for training. Model optimization and validation occurred on the Mayo dataset, tuning L2 regularization and learning rate. We evaluated metrics such as AUC, F1 score, SHAP values, and protein–pathway graphs. Finally, the pretrained model was tested independently on the Stanford dataset using the optimal hyperparameters.

### 2.3 Multi-omic APNet model

Using scMINER, we transformed count matrices from the scMGH and Blish PBMC scRNA-seq datasets into activity values (again avoiding data leakage). We identified common active molecules with the MGH plasma proteomic dataset and built a new one-hot encoded molecule–pathway matrix to train another APNet model on MGH activity values. Model optimization and validation were performed on the scMGH dataset (similar to the first scenario). The pretrained model from the MGH–scMGH datasets was independently tested on the Blish dataset, using its significant feature drivers that aligned with the model’s input features.

### 2.4 Brief description of APNet modular architecture

#### 2.4.1 Activity data preprocessing for DL input

For plasma proteomics, we used NetBID2 (Dong *et al.* 2023), while for scRNA-seq we used scMINER. These tools rely on the SJARACNe algorithm to reverse-engineer context-specific interactomes, capturing ‘activity values’ for each candidate driver (protein or gene). In bulk plasma proteomics, we consider all proteins as potential drivers for SJARACNe.

SJARACNe extends the original ARACNe approach (Margolin *et al.* 2006) for big data, focusing on whether mutual information or correlation potentials are significantly nonzero (Khatamian *et al.* 2019). NetBID2 uses SJARACNe outputs to compute driver ‘activity values’ by a weighted mean over each driver’s target proteins:

$${\text{Driver}}_{si}=\frac{\sum_{j=1}^{n}\left({\text{SIGN}}_{ij}\times {\text{MI}}_{ij}\times {\;\text{EXP}\;}_{sj}\right)}{n},$$

where ${\;\text{EXP}\;}_{sj}$ is the expression (e.g. NPX) of target protein j in sample s, ${\text{MI}}_{ij}$ is the mutual information between driver i and target j, and ${\text{SIGN}}_{ij}$ is the sign of their Spearman correlation. Finally, n is the number of targets regulated by i.

Differential activities (severe versus nonsevere) are computed via ‘getDE.BID.2G’. In single-cell data, scMINER similarly extracts *t*-test based differential activity (Ding *et al.* 2023).

#### 2.4.2 Biological priors via pathway enrichment

We mapped the joint differentially active severity drivers from the three Olink studies to biological pathways using the Enrichr KG (Evangelista *et al.* 2023). We leveraged 30 pathways each from KEGG, Reactome, GO: BP, and Wikipathways 2021 for up- and down-regulated drivers, then used one-hot encoding (1 if a driver belongs to a pathway gene set, else 0).

#### 2.4.3 DL Classification with biological priors

APNet’s DL classification uses a sparse neural network, PASNet (Hao *et al.* 2018), which incorporates gene/protein–pathway hierarchical connections, enabling more interpretable, explainable predictions. We tested 3 scenarios:

1. Two bulk-proteomic scenarios (MGH–Mayo, MGH–Stanford).
2. One single-cell scenario (MGH proteome–MGH scRNA-seq).

In each scenario, training occurred on the MGH dataset; we validated and tested on the other datasets. Activity matrices from NetBID2 or scMINER served as inputs, plus one-hot driver–pathway matrices as priors. Model performance was measured via AUC and F1, plus ROC curves.

Mathematically, PASNet enforces layer-wise sparsity and cost-sensitive learning. Let $W^{\left(l\right)}$ be weights at layer l, $M^{\left(l\right)}$ a mask matrix (pruning small weights), and λ a regularization hyperparameter:

$$h^{\left(l+1\right)}=a\left((W^{\left(l\right)}\;○\;M^{\left(l\right)})h^{\left(l\right)}+b^{\left(l\right)}\right),$$

where ○ is elementwise multiplication. For imbalanced data, the cost function includes class-specific average errors $C_{k}$. We optimize weights $W^{\left(l\right)}$ and biases $b^{\left(l\right)}$ via gradient updates:

$$W^{\left(l\right)}\leftarrow (1-\eta \lambda )W^{\left(l\right)}-\eta \sum_{k=1}^{K}\frac{\partial C_{k}}{\partial W^{\left(l\right)}},\;b^{\left(l\right)}\leftarrow b^{\left(l\right)}-\eta \sum_{k=1}^{K}\frac{\partial C_{k}}{\partial b^{\left(l\right)}}.$$

#### 2.4.4 Post-hoc explainable insights

APNet’s final step offers various graph-based visualizations and analytics to glean biological insights:

- *SHAP-based feature ranking:* SHAP values give feature-level interpretability (Hao *et al.* 2018).
- *Regulatory subgraphs:* We can isolate top-20 predictive drivers by SHAP values and reconstruct their local SJARACNe sub-networks.
- *Driver–pathway bipartite graphs:* Edges carry MI, correlation metrics (from SJARACNe), plus PASNet learning weights. Analysts can filter to identify potential translational subgraphs.
- *Shortest-path computations:* Using Dijkstra’s algorithm (via networkx) to find critical intermediates between two drivers of interest.
- *STRINGdb-based verification:* Because SJARACNe is unbiased, we can overlay known protein–protein interactions from STRINGdb to reduce spurious or unverified edges.

Formally, Shapley values ($\phi_{i}$) for feature i among M features are computed as:

$$\phi_{i}=\sum_{S\subseteq N\setminus \{i\}}\frac{|S|!(M-|S|-1)!}{M!}\left[f\left(S\cup \{i\}\right)-f(S)\right],$$

where f is our model and N the set of all input features (Hao *et al.* 2018).

### 2.5 Statistical metrics for data distribution analysis

During exploration of pre- and post-activity transformations, we assessed mean, standard deviation, kurtosis, and skewness for expression versus activity values (Montgomery and Runger 2014). Kurtosis gauges ‘tailedness’, skewness measures distribution asymmetry.

### 2.6 Clustering statistics during PCA analysis

To compare the 4 COVID-19 omic datasets (including MGH single-cell RNAseq) via PCA, we first converted scMGH data to pseudobulk to reduce complexity. We then computed:

- *Silhouette Score* (Rousseeuw 1987): from −1 to 1, higher is better separation.
- *Davies–Bouldin Index* (Davies and Bouldin 1979): lower is better; measures cluster similarity ratios.
- *Calinski–Harabasz Index* (Caliński and Harabasz 1974): higher is better; ratio of between- versus within-cluster variance.

### 2.7 Single-cell analysis and visualization

We used Scanpy (https://scanpy.readthedocs.io/) and Seurat v5 (https://satijalab.org/seurat/) for scRNA-seq. Seurat’s SCTransform function normalized the data matrix, after which *t*-tests compared expression in severe versus nonsevere groups.

### 2.8 Benchmarking and bioinformatic validation based on COVID-19 prior knowledge

PASNet baseline:

We built a baseline PASNet model using raw expression (rather than activity) from MGH, Mayo, and Stanford. Genes with significant differential abundance across these datasets were kept. As before, we used Enrichr-KG pathways and measured performance by AUC, F1, etc.

Random Forest baseline:

We also trained a Random Forest on activity matrices from the four datasets in two rounds:

1. Train on MGH proteomic, test on Mayo/Stanford.
2. Train on MGH proteomic, test on MGH single-cell data.

For each experiment, we took the top-20 predictive drivers (by SHAP or feature importance) and retrieved protein–protein interaction (PPI) networks from STRINGdb (score > 0.4). We computed assortativity and average node degree in each PPI (Newman 2002). We also mapped top drivers to nine curated COVID-19 immunopathology hallmarks from SIGNOR (Level 4 networks in Cytoscape).

### 2.9 DOME recommendations

APNet’s assembly adheres to DOME guidelines (Walsh *et al.* 2021) for reporting supervised ML analyses in biological contexts (see Supplementary Material S1).

## 3 Results

### 3.1 Harmonizing COVID-19 proteomic and transcriptomic datasets for APNet deployment

Initially, we harmonized patient stratification for COVID-19 severity based on WHOscore (‘Severe’ versus ‘NonSevere’) across the three Olink proteomic datasets. In particular, COVID-19 cases who had a fatal outcome or were admitted to the ICU or were intubated were designated as ‘severe’, and the remaining cases were defined as ‘nonsevere’. In the MGH study, we designated 80 severe and 225 non-severe cases. We designated 268 severe and 181 non-severe COVID-19 cases in the Mayo study. Furthermore, we determined 24 severe and 40 non-severe cases in the Stanford study. Associations with respective WHOscores and age can be seen in (Supplementary Fig. S1). Summary statistics of the three proteomic datasets regarding clinical covariates can be found in Supplementary Figs S1–S3. MGH contains extensive clinical features, including comorbidities (e.g. hypertension, diabetes, heart and kidney disease), biochemical markers (CRP, D-dimer, LDH), and patient demographics. Mayo and Stanford include more limited clinical information.

From all 3 Olink studies, 1463 common plasma proteins were bioinformatically studied within APNet and used for downstream processing to uncover predictive markers of COVID-19 severity (bulk scenario).

Corollary to these proteomic data, our study included two single-cell RNA-seq PBMC datasets by the Villani group (4 severe and 10 non-severe MGH cases, 6665 cells, 15575 genes) and the Blish group (4 severe and 4 nonsevere, 6500 cells, 16234 genes). The overarching goal here was to seek common signalling motifs on a multi-omic spectrum (i.e. across bulk plasma proteomics and PBMC scRNA-seq data), given that part of the plasma proteome is produced by circulating peripheral blood mononuclear cells (PBMCs) involved in COVID-19 immunopathology (single-cell scenario) (Supplementary Fig. S4).

### 3.2 APNet aligns data distributions, mitigates batch effects and increases the breadth of jointly regulated drivers after activity transformations

At this point, we deployed the NetBID2 tool on the bulk plasma proteomic datasets and the scMINER tool on the PBMC scRNA-seq datasets to attain the activity-transformed omic matrices which will be used subsequently for the analyses (see Materials and Methods).

Once the activity transformations were complete, data exploration was conducted to document the extent of alterations in the datasets after pre-processing with NetBID2/ScMINER. Noticeably, the distributions of the activity values for the bulk proteomics across the three datasets aligned almost entirely (Mean: 0.32, 0.32, 0.45 and Standard Deviation: 0.12, 0.15, 0.16 for MGH-Mayo-Stanford respectively), which was not the case for the distributions of the expression values (Mean: -0.74, 1.56, 4.88 and Standard Deviation: 1.48, 1.69, 2.38 for MGH-Mayo-Stanford respectively) (Supplementary Table S1) (Supplementary Figs 5A–C and 6A–C). Similarly, pre-processing of MGH scRNA-seq data with scMINER almost doubled the mean values and standard deviations of the distribution of activity values compared to those of expression values, entailing negative values. Focusing on the positive values, the two distributions differed significantly in kurtosis and skewness (0.76 and 0.88 for activity; 5.32 and 2.02 for expression), which indicated that the positive activity values had fewer outliers and were more evenly distributed than the expression ones. A similar trend was observed also in the Blish scRNA-seq dataset, post-scMINER (Supplementary Table S2) (Supplementary Fig. S7). Overall, these results suggested that the activity transformations aligned data distributions from COVID-19 bulk proteomics and scRNA-seq data (by increasing the ‘signal-to-noise’ ratio in the latter since more genes acquired stronger activity signal), paving the way for more accurate multi-omic DL predictions (Supplementary Table S1).

**Figure 2.**
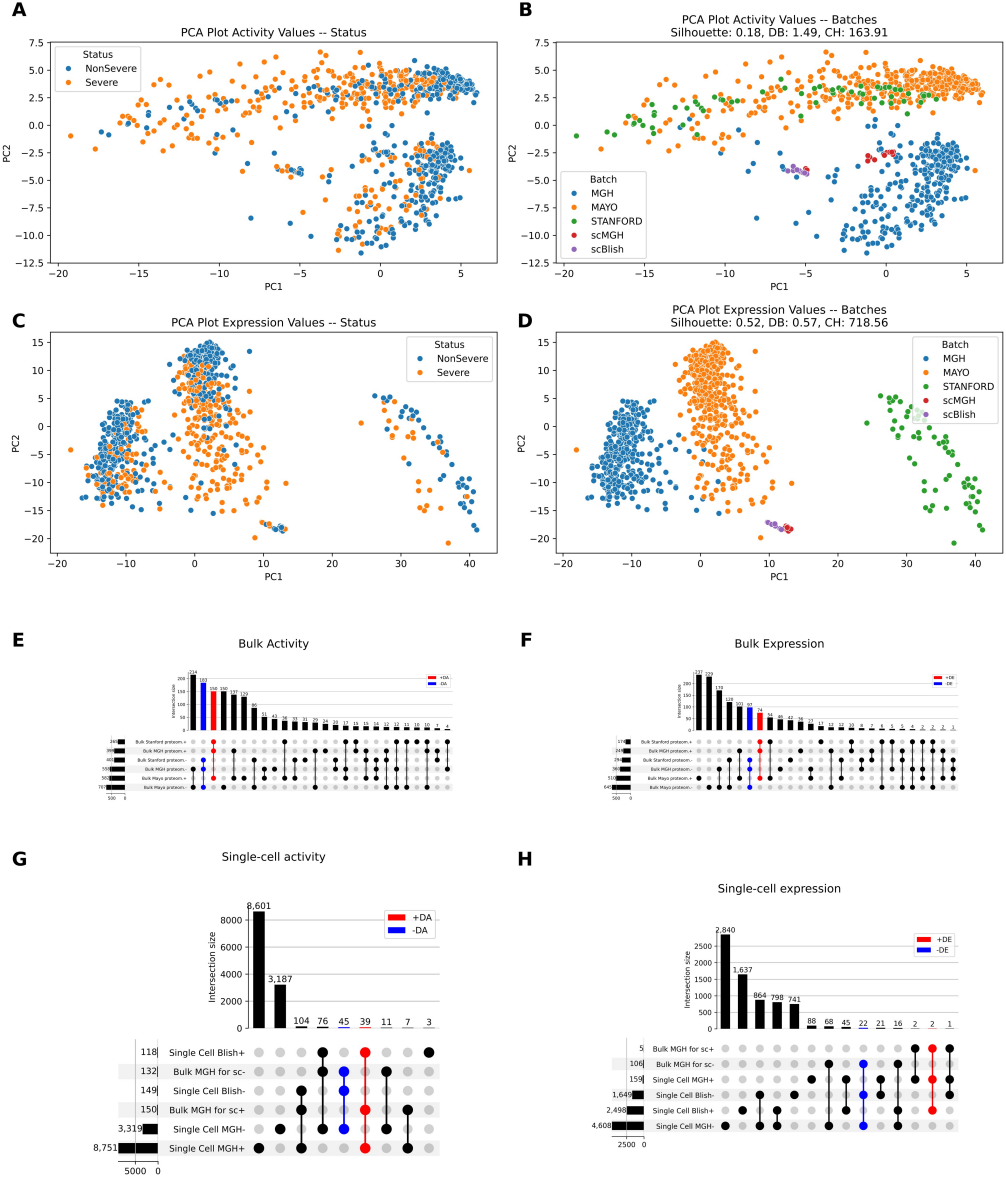
APNet harmonizes diverse data distributions, reduces batch effects, and broadens the range of joint differentially active drivers. (A) PCA plot illustrating data separation with activity values, colored by Severe versus NonSevere. (B) Same as (A) but colored by dataset. (C) PCA plot with expression values colored by Severe versus NonSevere. (D) Same as (C) but colored by dataset. (E) UpSet plots for the common significant proteomic drivers (MGH, Mayo, Stanford) using activity. (F) Same as (E) but with expression. (G) UpSet plots for differentially active drivers across MGH, scMGH, and Blish. (H) Same as (G) but with expression values.

Next, we performed PCA analysis with calculations of clustering statistics (Silhouette Score/Silh.S.; Davies-Bouldin Index/DBI; Calinski-Harabasz Index/CHI) on the activity and expression matrices of the four COVID-19 datasets (scRNA-seq were included after pseudo-bulk calculation—see Section 2). Activity transformations ameliorated the batch effect across the four studies and attenuated data clustering, as demonstrated by the lower Silh.S. and CHI and higher DBI (0.18, 163.91, 1.49) compared to the corresponding outcomes from expression matrixes (0.52, 718.56, 0.57) (Fig. 2A–D).

After batch effect inspection, the differentially active proteins and genes (DAPs, DAGs) and their differentially expressed counterparts (DEPs, DEGs) were calculated for the severe and nonsevere COVID-19 cases across the four studies.

Strikingly, the activity transformations led to the detection of 333 jointly DAPs (150 differentially hyper-active and 183 hypo-active) across the 3 Olink bulk plasma proteomic studies, in contrast to the 163 proteins indicated by typical differential abundance analysis (Fig. 2E and F). Cumulative logFC plotting across the three studies revealed that activity analysis prioritized plasma proteins distinct from those obtained from expression analysis, in terms of which molecules had the higher magnitude of differential change in severe COVID-19 (Supplementary Figs S5D–G and S6D–G). Consequent enrichment of implicated biological processes (GO: BP) revealed that activity analysis ranked terms related to inflammatory viral infection (high logFC drivers, Supplementary Fig. S5H) and the systemic multi-organ failure (low logFC drivers, Supplementary Fig. S5I) at the top positions compared with the respective enrichment analysis of expression matrices (Supplementary Fig. S6H and I).

APNet captured 282 differentially active drivers between the MGH plasma proteomic and scRNA-seq datasets, contrary to the 140 joint drivers detected by the differential abundance analysis (Fig. 2G and H). Cumulative logFC plotting showed again divergent results between activity and expression values in this scenario. Ensuing pathway enrichment revealed that top logFC drivers based on activity gravitated towards metabolic alterations for hyper-active drivers and immunological defects, especially for T-cells, for hypo-active drivers compared to expression analysis (Supplementary Fig. S8).

In the bulk plasma proteomic scenario, the 333 joint DAPs were almost equally categorized in overt (170) and hidden drivers (163). MGH was the one that had the highest fraction of hidden drivers (111/333), followed by Stanford (81/333) and Mayo (23/333) (Supplementary Fig. S9A). In the bulk/single-cell scenario, across the three datasets MGH Olink, MGH scRNAseq, and Blish scRNAseq, most common drivers were hidden (71/85), and their percentage in bulk and MGH/Blish scRNA-seq datasets approximated almost 40%, 36%, and 53%, respectively (34/85, 31/85, and 45/85 respectively) (Supplementary Fig. S9B).

### 3.3 Pathway enrichment on APNet’s severity drivers reveals hallmarks of COVID-19 immunopathology

In the second part of the APNet framework, pathway enrichments of the common hyper-active and hypo-active DAPs across the three proteomic datasets were conducted using Enrichr KG (https://maayanlab.cloud/enrichr-kg) to create biological priors for the MGH–Mayo and MGH–Stanford training/validation/testing scenarios that would ensue. A similar approach was also followed for the bulk/single-cell training/testing scenario, entailing common hyper-active and hypo-active DAPs/DAGs between the bulk MGH proteomic dataset and the MGH PBMC scRNA-seq dataset.

Based on the Enrichr combined score, the enrichments for the hyper-active drivers revealed pathways documented in the literature as COVID-19 ground-truths like COVID-19 adverse effects, neuroinflammatory responses, apoptosis, viral infection, deregulation in NAD^+^ metabolism, lung fibrosis, atherosclerotic incidents, and aberrant interleukin signalling (Supplementary Fig. S10A and C). On the contrary, the analysis of the hypo-active drivers showed opposite trends for pathways concerning cell adhesion, heart morphogenesis, organization of the extracellular matrix, B and T cell function, and hematopoietic stem cell homeostasis (Supplementary Fig. S10B and D).

### 3.4 Explainable APNet models robustly classify severe and nonsevere COVID-19 in bulk proteomic and scRNA-seq scenarios

Next, we used the common DAPs across the three Olink plasma proteomic studies to predict severe versus nonsevere COVID-19 cases. During the MGH–Mayo experiment (initial training and model optimization-evaluation), APNet accurately predicted severe COVID-19 patients with significant robustness (AUC = 0.96, F1-score = 0.89). During the testing phase, the pre-trained bulk proteomic APNet model emerged as a performant classifier for COVID-19 severity on the Stanford dataset (AUC = 0.91, F1-score = 0.68).

**Figure 3.**
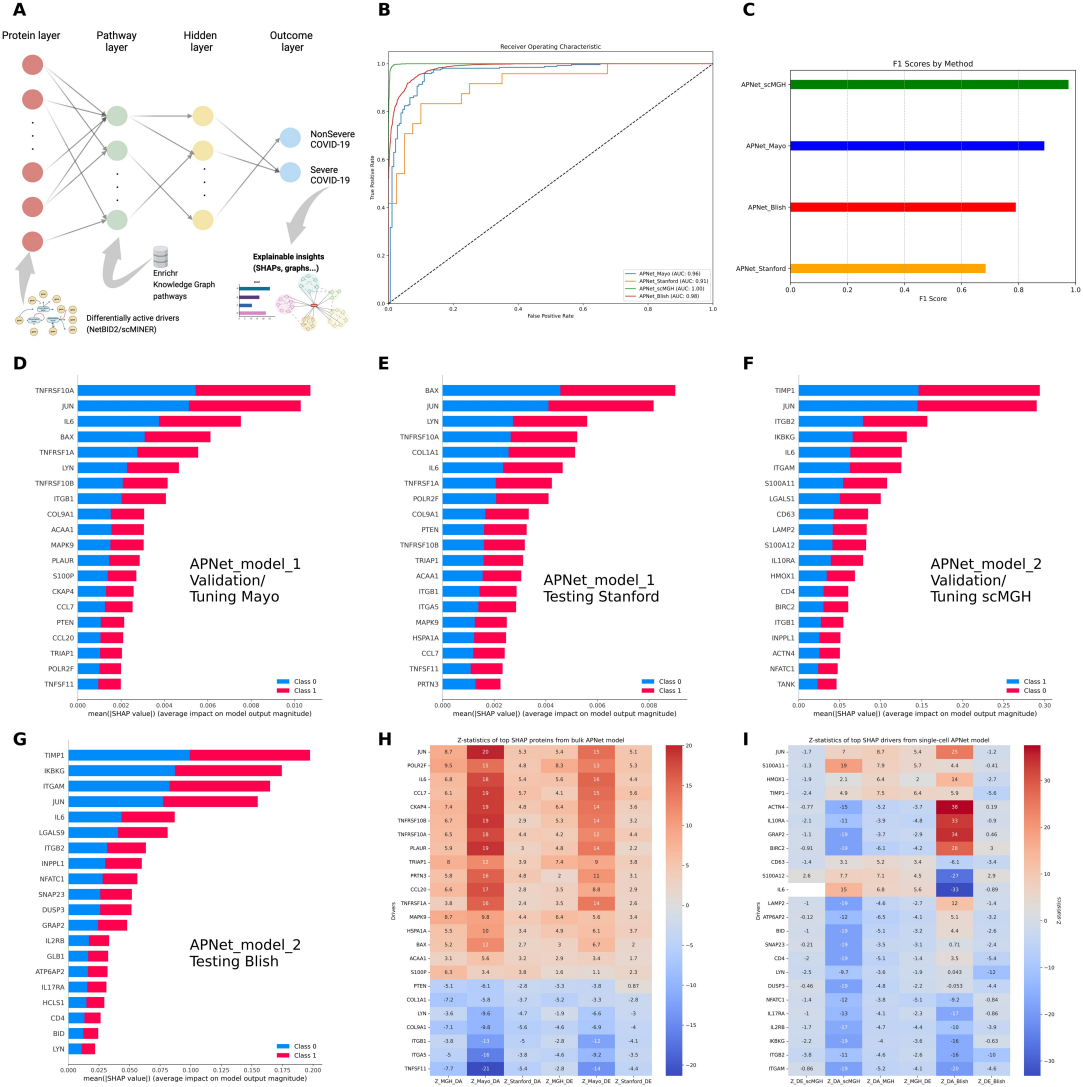
Explainable APNet classifies robustly severe and nonsevere COVID-19 cases in bulk proteomic and scRNA-seq scenarios. (A) An overview of the APNet pipeline with an emphasis on the biologically informed and explainable neural network component from PASNet. (B and C) ROC curves showing the AUC scores (B) and barplots showing the F1 score (C) for the various APNet deployments (MGH-Mayo training-optimization/tuning and Stanford testing, MGH-scMGH training-optimization/tuning and Blish testing). (D and E) The top 20 SHAP values from APNet models. (F) Bulk MGH-Blish scRNA-seq datasets. (H and I) Heatmaps displaying the *Z* scores for differential activity (DA) and expression analyses (D and E) for the top SHAP proteins across various APNet deployments. Values between −20 and 20 are considered statistically nonsignificant, given that the degrees of freedom (df) are set to 30 and the significance level (alpha) is 0.05 for a two-tailed test.

Subsequently, we created a new multi-omic APNet model, trained on MGH plasma proteomics and optimized/evaluated on the scMGH dataset, which exhibited strong performance in classifying severe COVID-19 cases (AUC: 0.99, F1-score: 0.975). This new pre-trained model was also robust during its independent testing phase with the Blish dataset (AUC: 0.98, F1-score: 0.79) (Fig. 3A–C).

Regarding the most predictive drivers of severity, the first APNet model, through top SHAP values, highlighted severity drivers involved in members of the TNF family (TNFRSF10A, TNFRSF10B, TNFRSF1A, TNFSF11), the transcription factor JUN, cytokines/chemokines (IL6, CCL7), apoptosis regulators (BAX, TRIAP1, PTEN), cell adhesion and ECM regulators (ITGB1, ITGA5, COL9A1, COL1A1, PLAUR, PRTN3), the stress response chaperone HSPA1A, signal transducers (MAPK9, LYN, POLR2F), and immuno-metabolic regulators (ACAA1, CKAP4, S100P). Notably, JUN, TNFRSF10, BAXA, and LYN were predictive plasma proteins, while ACAA1, BAX, LYN, PLAUR, PRTN3, PTEN, and S100P were also distinguished (Fig. 3D and G).

On the other hand, the biological explainability of the second APNet model revealed some divergent results based on SHAP values, possibly due to its multi-omic nature. The top-predictive drivers included various biological motifs, such as signal transduction (JUN, IKBKG, IL6, NFATC1, LYN, GRAP2), immune response and inflammation (IL6, ITGAM, ITGB2, IL2RB, IL10RA, IL17RA, CD4, TIMP1), extracellular matrix remodelling (TIMP1, ITGAM, ITGB2, ACTN4, LAMP2), stress response and apoptosis (BID, BIRC2, HMOX1, DUSP3, ATP6AP2), vesicle trafficking (SNAP23, LAMP2, CD63), and calcium signalling (S100A11, S100A12). Of note, most of these multi-omic drivers were hidden drivers of COVID-19 severity (ACTN4, ATP6AP2, CD4, DUSP3, GRAP2, HMOX1, IKBKG, IL10RA, IL17RA, IL6, JUN, LAMP2, LYN, NFATC1, S100A11, and SNAP23) (Supplementary Fig. S11).

### 3.5 APNet connects predictive proteins to predictive pathways, revealing clinically relevant motifs

After identifying highly predictive drivers in bulk proteomic and single-cell scenarios, APNet’s enhanced explainability was utilized to gain insights into biological pathways predictive for COVID-19 severity. This approach is based on the rationale that drivers involved in common pathways likely fall into co-regulation, which APNet can identify, revealing significant predictive biological motifs.

**Figure 4.**
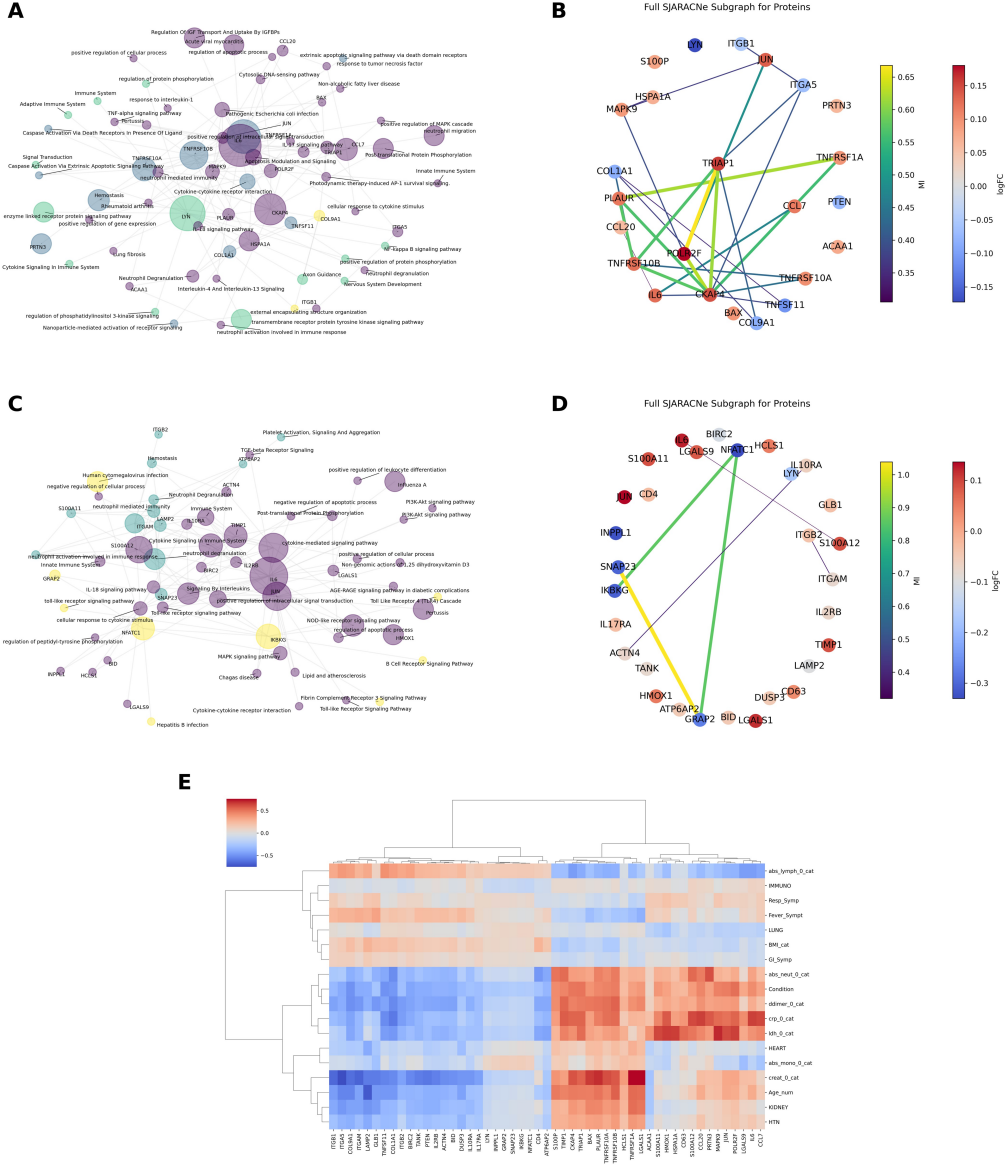
APNet connects predictive drivers of COVID-19 severity with predictive pathways and offers clinico-biological insights. (A) Protein–protein and protein–pathway bipartite graphs for the top 20 most predictive drivers (SHAP values, SJARACNe regulatory networks) with their highly predictive pathways (PASNet learning weights), for the Model 1 scenario; node color is determined based on network communities, while node size is based on betweenness-centrality. (B) SJARACNe regulatory subgraph focusing on the top 20 predictive drivers, where nodes are colored by logFC and weights by SJARACNe MI value. (C–D) Same as (A) and (B) but for the Model 2 scenario. (E) Pearson correlation heatmap for top SHAP predictive drivers and clinical covariates from the MGH dataset; these covariates are established clinical markers associated with severe COVID-19.

In Fig. 4A and B, the explainability of the bulk pre-trained APNet model (MGH training–Mayo validation/parameter tuning–Stanford independent testing) is showcased by a bipartite protein–pathway network assembled from (a) the proteomic MGH SJARACNe regulatory network of the most predictive SHAP protein drivers and (b) predictive driver–pathway connections from the Enrichr Knowledge Graph (KG) mappings, thresholded by the highest APNet learning weights assigned during the MGH–Mayo training–validating stages.

Apart from S100P, PTEN, ACAA1, BAX, and LYN, all other predictive drivers participated in a single SJARACNe regulatory subgraph, with CKAP4, POLR2F, and TRIAP1 sharing the most substantial connection in terms of MI (> 0.6) (Fig. 4A and B). The bipartite APNet graph contained a large community connecting several drivers (IL6–JUN–CCL7–MAPK9–POLR2F–HSPA1A–BAX–TRIAP1), associated with interleukin signalling (IL4/13/17/18), cytokine stimulation, liver disease, and lung fibrosis. A smaller community connected ACAA1, CKAP4, PLAUR, and TNFSF11 with neutrophil activation and inflammation. Meanwhile, LYN formed a small subgraph with the PI3K signalling pathway, adaptive immune system, NF-κB pathway, and neurotoxicity. Another small cluster contained TNF family proteins (TNFRSF1A/10A/10B) and caspase-induced apoptosis. Overall, these results indicated the destructive nature of severe COVID-19 tied to systemic organ failure.

Lastly, in the bulk/single-cell scenario, no SJARACNe regulatory subgraph was found among these predictive drivers, apart from a ‘network island’ of NFATC1, IKBKG, GRAP2, and SNAP23 and smaller dyadic subgraphs of ITGAM–ITGB2, IL6–S100A12, and ACTN4–IL10RA. The respective APNet bipartite graph contained a large cluster (IL10RA, ACTN4, ITGB2, IL2RB, BIRC2, ITGAM, HMOX1, TIMP1) linked with established COVID-19 inflammatory and immune signalling cascades. Other noticeable network clusters included NFATC1–GRAP2 (viral infection), IL6–S100A12 (neutrophil activation), and IKBKG (complement and B cell activation). Interestingly, no SJARACNe regulatory subgraph was obtained among these predictive drivers apart from a ‘network island’ of NFATC1, IKBKG, GRAP2, and SNAP23 and smaller dyadic subgraphs of ITGAM–ITGB2, IL6–S100A12, and ACTN4–IL10RA (Fig. 4C and D).

To corroborate that these predictive drivers and, by extension, their neighbouring pathways bear clinical connotations, Pearson correlations were calculated among these top SHAP drivers and the MGH clinical covariates. The hyper-active drivers in severe COVID-19 exhibited strong positive correlations with typical adverse prognosticators, such as increased D-dimer, CRP, LDH, absolute neutrophil count, and specific comorbidities (kidney disease, hypertension). By contrast, the hypo-active drivers had negative correlations with those clinical covariates and showed a positive correlation with absolute lymphocyte count (Fig. 4E).

Overall, during the DL predictions, APNet efficiently connected predictive drivers to pathways deemed relevant to the clinical course of COVID-19. Supplementary Table S2 summarizes the implications of such pathways in COVID-19 immunopathology, citing relevant publications from the literature and underlining the relevance of APNet’s analysis in this disease context.

### 3.6 APNet reveals underlying biological motifs in severe COVID-19: the case of ACAA1

To showcase APNet’s capacity to expose non-obvious connections among clinically predictive drivers and pathways, we investigated how ACAA1 (acetyl-Coenzyme A acyltransferase 1), a key regulator of fatty acid β-oxidation in peroxisomes and a highly predictive COVID-19 severity driver (based on APNet from the bulk proteomic scenario), is connected to other equally predictive proteomic drivers. ACAA1 was also selected because it shared a direct SJARACNe co-regulatory connection with ACE2, the cardinal molecule for SARS-CoV-2 viral tropism.

**Figure 5.**
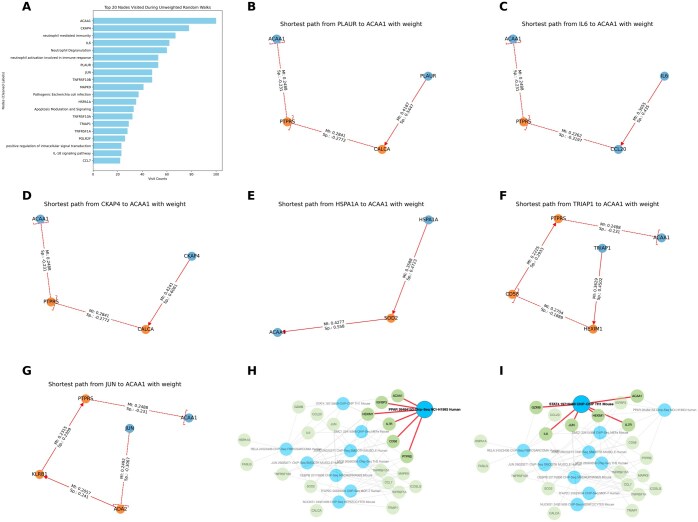
APNet enables the assembly of complex graphs that can be leveraged to discover nonapparent connections of ACAA1 with other predictive drivers of COVID-19 severity. (A) Barplot displaying the number of visits during random walks initiated using the NetworkX package, starting from ACAA1 in the driver-pathway bipartite graph generated by the bulk APNet model. (B–G) Based on Dijkstra’s algorithm, several shortest paths from top SHAP predictive drivers to ACAA1, with Spearman correlation coefficients (positive = activation, negative = inhibition) as edge weights. (H and I) Enrichr KG gene-transcription factor bipartite graphs leveraging information from the Chea3 database concerning ACAA1, top SHAP drivers, and intermediate proteins based on the bulk proteomic scenario of APNet. These Enrichr KG graphs serve as independent biological evaluation of the aforementioned APNet shortest paths from top SHAP drivers towards ACAA1.

When random walks were initialized from the ACAA1 node on the pertinent APNet driver–pathway bipartite graph (Fig. 4A), several top-20 SHAP drivers (CKAP4, IL6, PLAUR, TNF family proteins, JUN, MAPK9, TRIAP1, HSPA1A, POLR2F, CCL7) emerged as highly visited nodes, along with nodes representing pathways of neutrophil hyper-activation (Fig. 5A).

Using Dijkstra’s algorithm with the aforementioned SHAP proteins as sources (and ACAA1 as the target), we captured several intermediate proteins involved in immune and inflammatory responses (CCL20, IL7R, CD58, ICOSLG), apoptotic regulation (FASLG), and stress response (SOD2). Most of the shortest paths reached ACAA1 through the neuro-hormonal mediator PTPRS, a negative co-regulator of ACAA1 (Fig. 5B–D, F, and G). Only one path (successive positive co-regulations) reached ACAA1 via a different immediate neighbour (HSPA1A → SOD2 → ACAA1) (Fig. 5E). To validate the directionality rationale for considering ACAA1 as the target, we applied the GENIE3 algorithm to the top SHAP plasma proteins identified by the bulk APNet model. The analysis confirmed that other top SHAP proteins indeed propagate signals toward ACAA1. Notably, HSPA1A emerged as the most critical regulator influencing ACAA1, followed by MAPK9, ITGA5, POLR2F, TNFRSF1A, and ITGB1 (Supplementary Fig. S12).

To evaluate the credibility of the retrieved shortest paths, we hypothesized that clinical drivers participating in plausible shortest paths might reflect common underlying molecular regulation. For this purpose, we queried the Enrichr KG with ACAA1, source proteins, and intermediates, focusing on the Chea3 database (https://maayanlab.cloud/chea3/) for transcription factor enrichment. Notably, two ACAA1-related transcription factors, PPAR and STAT4, emerged as significant, each connecting to HEXIM1/IL7R and other distinct drivers: IGFBP3–CD58–PTPRS (PPAR) and GZMB–IL6–JUN (STAT4). Considering that STAT4 participates in interferon signalling hyperstimulated during SARS-CoV-2 infection and the implications of peroxisome proliferator-activated receptors (PPARs) in COVID-19 hyperinflammation (Lee *et al.* 2020, Hasankhani *et al.* 2023), these results further corroborate the biological relevance of APNet’s insights regarding the broader involvement of ACAA1 and other highly predictive plasma proteins in severe COVID-19 pathobiology (Fig. 5H and I).

### 3.7 APNet outperforms alternative ML/DL classifiers in predicting severe COVID-19 cases

To benchmark APNet’s robust performance on classifying severe COVID-19 cases, we initially retrieved from the literature the predictive models published by the authors of the MGH study (Filbin *et al.* 2021), the Stanford study (Feyaerts *et al.* 2022), and from an independent study in Qatar (which used MGH for external validation). As shown in Table 1, APNet outperformed both the MGH and Stanford models. Although APNet showed similar performance to Qatar’s predictive model (AUC > 0.95, training/testing on the authors’ in-house data) in demarcating severe COVID-19 cases, it outperformed Qatar’s model in terms of generalizability. Indeed, the latter achieved an AUC of 0.79 when independently tested on the MGH study (Table 1).

**Table 1.** Published ML/DL analyses of MGH and Stanford Olink datasets.^a^

Study	AI model	AUC
MGH study	Random Forest	0.85
Stanford study	(LASSO) linear regression	0.77–0.79 (Stanford study)
Qatar study	MUVR	>0.959 (Qatar data), 0.76 (D0) (MGH validation)

a This table shows previously published models for COVID-19 severity classification.

At this point, we performed more specific benchmarking experiments using:

1. Normalised expression values of COVID-19 plasma proteomics, omitting any activity transformations with a PASNet model (*BenchPASNet*);
2. Activity values of COVID-19 plasma proteomics and scRNA-seq but with a Random Forest (RF) classifier (*BenchRF*).

**Figure 6.**
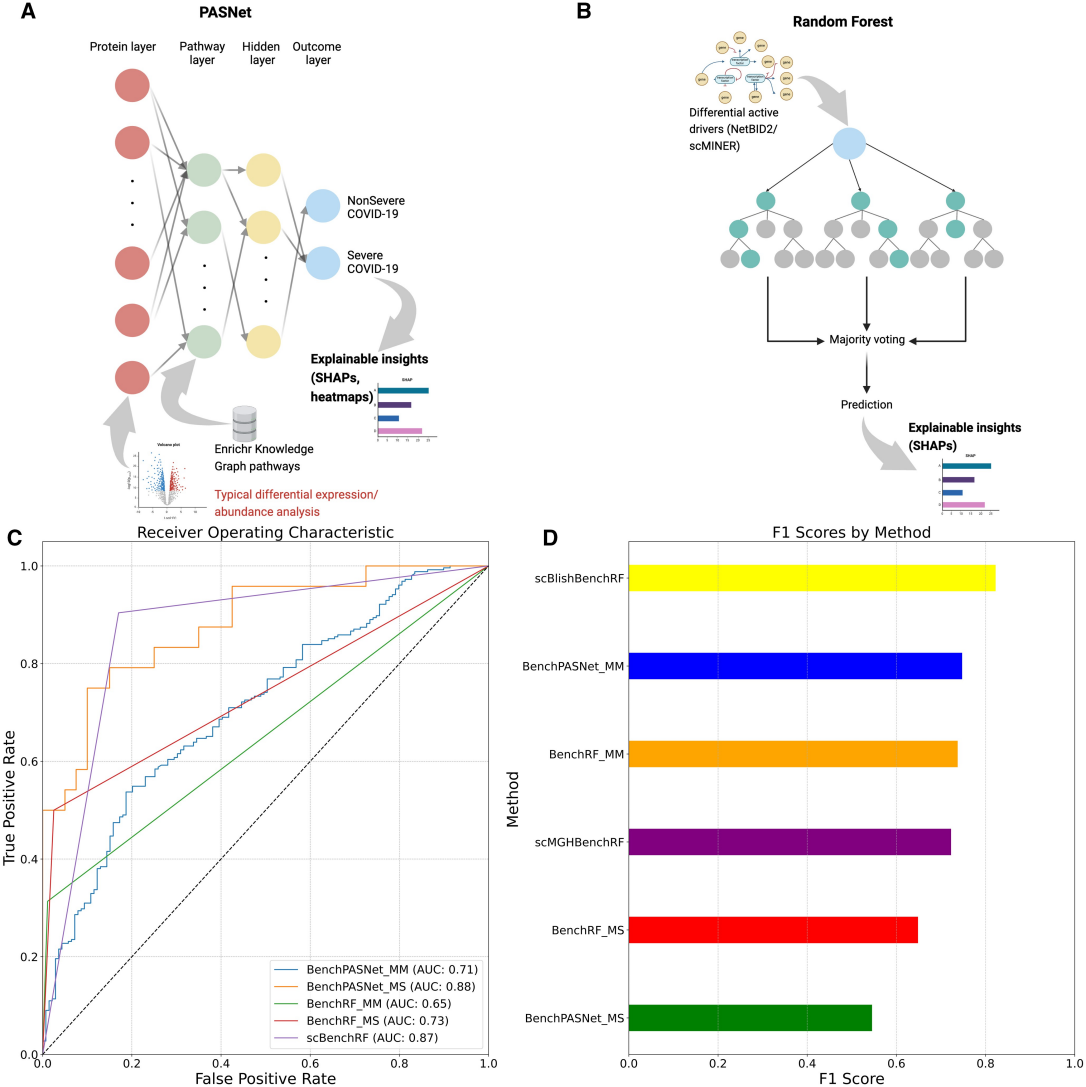
APNet outperforms alternative models for severe COVID-19 predictions. (A and B) Schematic representations of BenchPASNet (A) and BenchRF (B) approaches competing with APNet. (C and D) ROC curves with the AUC scores (C) and barplots showing the F1 score (D) across various benchmarking approaches.

The training, validation, and testing datasets remained the same (Fig. 6A and B).

Notably, all alternative models underperformed relative to APNet in predicting severe COVID-19. Specifically, BenchPASNet performed poorly in the MGH–Mayo scenario (AUC: 0.71, F1-score: 0.78), although slightly better in MGH–Stanford (AUC: 0.88, F1-score: 0.54), and it failed to identify hidden drivers. Strikingly, the RF-based model underperformed even more in both scenarios (AUC: 0.65, F1-score: 0.73 for MGH–Mayo; AUC: 0.47, F1-score: 0.65 for MGH–Stanford) (Fig. 6C and D).

For the multi-omic experiment, we did not test a PASNet expression-driven model due to the intrinsic sparsity of scRNA-seq expression and the need for complex data harmonization beyond our project scope. Instead, we used RF on the shared perturbational space of plasma proteomics and scRNA-seq activity data. This approach again underperformed APNet’s single-cell model, achieving AUC: 0.87, F1-score: 0.73 (MGH–scMGH) and AUC: 0.64, F1-score: 0.80 (MGH–Blish) (Fig. 6C and D).

Regarding the top-20 most predictive drivers across all experiments, there were significant deviations between APNet runs and other benchmark models (Supplementary Fig. S13), as shown by the Supervenn plot (Supplementary Fig. S14). Only 10 proteins appeared in four or more experiments among all top SHAP drivers (98) from the eight total models tested.

### 3.8 APNet outperforms alternative ML/DL classifiers in capturing COVID-19 biological ground truths

In this final part, we evaluated the potential of each model to recapitulate the biological ground truths associated with severe COVID-19 and provide biological insights into the outperformance of APNet in clinical predictions over the rest ML/DL models.

Primarily, we assembled driver-pathway bipartite graphs from BenchPASNet deployment on MGH-Mayo and MGH-Stanford scenarios and discovered that inflammation and neutrophil activation were the most predictive pathways in these benchmarking models. Compared to APNet results (Fig. 4), these benchmarking models could only partially capture clinically significant pathways, which could explain their inferior performance as severe COVID-19 classifiers (Supplementary Fig. S15).

**Figure 7.**
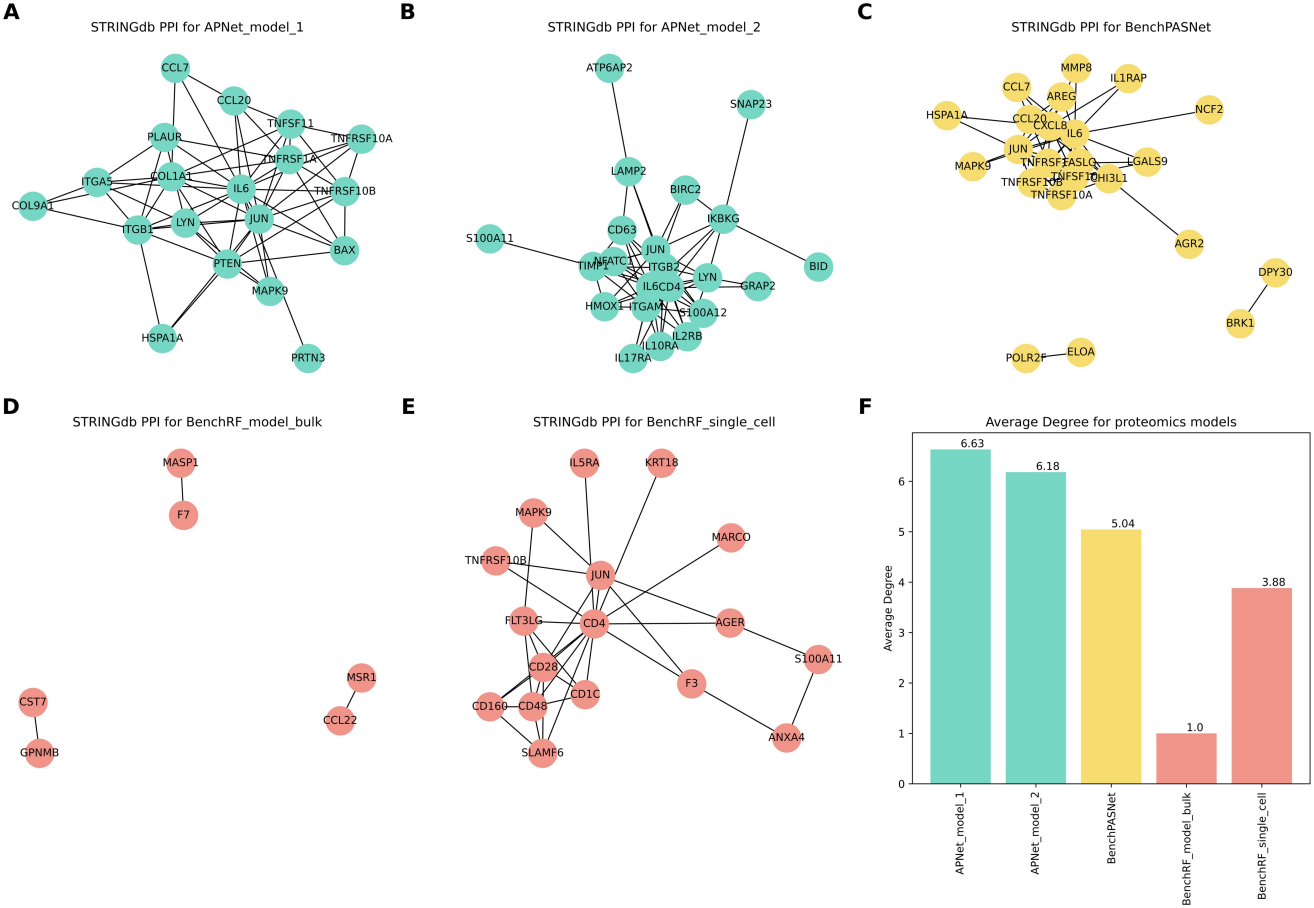
APNet prioritizes predictive drivers in severe COVID-19 that better capture underlying biological ground-truths. (A–E) STRINGdb protein–protein interaction (PPI) networks of the most predictive drivers based on SHAP values from the various APNet and benchmark models. APNet_model_1: APNet on the MGH-Mayo-Stanford datasets. APNet_model_2: APNet on the MGH-scMGH-Blish datasets. BenchPASNet: Original PASNet on bulk proteomic expression (NPX) values. BenchPASNet_MS: Original PASNet on MGH-Stanford expression values. BenchRF_model_bulk: RF classifier on bulk proteomic datasets. BenchRF_single_cell: RF classifier on bulk/single-cell experiments. (F) Average degree scores from STRINGdb networks are shown in (A–E). Network statistics were calculated using the NetworkX package in Python.

Furthermore, STRINGdb PPI networks were reconstructed for each group of top predictive drivers from each APNet and benchmark model, and specific network metrics to evaluate the degree of the biological content captured in each instance were calculated. We focused on the average node degree (the higher it is, the denser the network is in terms of connections) as the initial metric of choice. The APNet STRINGdb graphs in the bulk proteomic scenarios exhibited higher average node degree than the BenchPASNet STRINGdb graph, hence exhibiting higher interconnectivity among more homogenous nodes in terms of degree distribution. The differences were even more pronounced when comparing APNet STRINGdb graphs with their BenchRF counterparts since the latter were significantly smaller in size (Fig. 7). Furthermore, APNet’s STRINGdb graphs contained an equal or larger number of biologically significant edges (i.e. edges with STRINGdb combined score > 0.6, almost on par with the BenchPASNet, outperformance of RF models). Interestingly, the distribution of the edge weights were much more uniformal in the APNet models (closer to Gaussian distribution based on kurtosis-skeweness and log-likehood score) compared to the BenchPASNet which followed a bimodal distribution with many edges close 0,8–0,9 combined STRINGdb score and to the opposite end of 0,4 (Supplementary Table S4, Supplementary Fig. S16).

Next, we evaluated the correlations between the most predictive drivers from each benchmarking experiment with the clinical covariates of MGH. Unlike the APNet predictive drivers, the Pearson correlation heatmaps from the hierarchical clustering showed less distinct correlation profiles. These results indicated that the top predictive drivers from the benchmark experiments did not correlate with established adverse prognosticators as clearly as the APNet ones (Supplementary Fig. S17).

Lastly, to evaluate the degree of COVID-19 ground truths that APNet and the other classification models recovered, we mapped each model’s top 20 most predictive proteins from the various experiments to the SIGNOR 3.0 COVID-19 Hallmark pathways (i.e. Virus Entry, Cytokine storm, Inflammation, Fibrosis, Apoptosis, Innate response to dsRNA, MAPK Activation, ER stress and Stress granules,https://signor.uniroma2.it/covid/). The top SHAP predictive drivers from the APNet models exhibited a higher number of edges within the following curated networks (dsRNA-response, granules, fibrosis, inflammation) compared to the SHAP proteins from the benchmark models but the most stark difference was spotted in the apoptosis graph (Supplementary Table S3) (Supplementary Fig. S18).

## 4 Discussion and conclusion

In the herein work, we present APNet, a novel interpretable DL framework that is oriented towards supervised patient classification and explainable biological insights. To do so, it draws inspiration from two pillars of current DL trends in Systems Biology, which are biologically informed neural networks and DL models that entail extensive post-hoc explainability (Sapoval *et al.* 2022, Santorsola and Lescai 2023, Wysocka *et al.* 2023).

Regarding the first pillar, APNet ensures that the biologically informed DL component (PASNet model) ingests activity values of pre-processed omic datasets as data input and the protein/gene-to-pathway connections as biological priors (Hao *et al.* 2018). The concept of activity (NetBID2/scMINER tools, SJARACNe algorithm) goes beyond the static snapshot that typical differential expression analysis provides, as it captures linear and nonlinear molecular interactions, like post-translational modifications, in which the cause-and-effect relationships can arise at different time points and sub-cellular compartments (Wang *et al.* 2021, Ding *et al.* 2023). Hence, APNet guides the PASNet model to make predictions upon the post-activity transformations of omic data which: (a) align the distribution of diverse omic datasets and mitigate potential batch effects—two paramount factors in DL data pre-processing, (b) reflect the regulatory relationships among the various proteins and genes more reliably and (c) generate more biologically accurate driver-pathway mappings.

For post-hoc explainability, APNet offers a dynamic graph-based approach that incorporates SHAP values, driver-driver and driver-pathway connections, various node attributes (e.g. logFC of differential activity) and edge attributes (e.g. Mutual Information or Spearman correlation statistics from the SJARACNe algorithm, learning weights of driver-pathway connections from PASNet model). Network analysis is a significant asset for systems biology analysis since it is visually tractable by humans and can reveal biological motifs that connect diseases, pathways, omics, and patients. APNet provides a wide variety of graph visualization and analytical tools via the Networkx package, like clustering and shortest path discovery, which are critical in discovering obscure patterns across multi-omics (Zitnik *et al.* 2024).

In this work, we showcase the functionality of APNet in predicting severe from nonsevere COVID-19 cases across five different studies (3 bulk plasma proteomics 2 ancillary PBMC scRNA-seq datasets). Following DOME recommendations, APNet was trained and validated on separate datasets to ensure generalizability and avoid overfitting. APNet successfully identified severe COVID-19 cases by capturing well-known and hidden drivers along with ground-truth signalling pathways of severe COVID-19 immunopathology (Supplementary Table S2). APNet’s cross-modal prediction capabilities were noteworthy, as it identified similar pathways in bulk proteomic and in PBMC scRNA-seq data (e.g. neutrophil activation, T cell deregulation), without the need for pseudo-bulking for the latter case, hence preserving cellular heterogeneity (You *et al.* 2023). Furthermore, APNet uncovered nascent connections among ACAA1 and other predictive plasma proteins for severe COVID-19, like IL6 (Pro-inflammatory cytokine), CKAP4 (Cytoskeleton-Associated Protein involved in PI3K pathway), HSP1A1 (stress-induced Heat Shock Protein), and PLAUR (Plasminogen Activator—Urokinase Receptor, extracellular matrix regulator and tissue remodeller) through pertinent proteins (e.g. PTPRS), transcription factors (PPAR, STAT4) and pathways which reflect important COVID-19 hallmarks: lipid peroxidation, bioenergetic-metabolic deregulations, aberrations in the extracellular matrix and immune signalling impairments (Wang *et al.* 2023, Kamdar *et al.* 2024). Considering that ACAA1 was recently associated with high rates of Intensive Care Unit (ICU) admittance for patients with severe COVID-19 (Penrice-Randal *et al.* 2022), this outcome highlights the translational potential of APNet to uncover nuanced driver motifs with significant theragnostic value.

APNet outperformed published models in the field of COVID-19 plasma proteomics, but also alternative models that operated on the expression values of omic datasets (BenchPASNet) or used an RF classifier (BenchRF) instead of PASNet. APNet outperformed not only in terms of accuracy and robustness but also in capturing the biological ground truths associated with severe COVID-19. Compared to the benchmarking models, APNet prioritized the drivers that shared stronger STRINGdb interactions and connected them with biological pathways highly representative of COVID-19 immunopathology, as shown from our focused SIGNOR analysis (i.e. apoptosis). The distribution of STRINGdb combined scores in APNet networks displayed a more unimodal pattern (with a variety of STRINGdb combined scores ranging from 0.4 to 0.9), in contrast to the sharply bimodal distribution observed in the STRINGdb BenchPASNet network. This difference may reflect APNet’s inherent ability to capture a broader range of connections, enabling the identification of more subtle biological motifs for severe COVID-19.

Additionally, driver–pathway bipartite networks derived from the BenchPASNet indicated only a small number of implicated pathways (cytokine storm, neutrophil degranulation), whereas APNet revealed a broader set of pathways with highly predictive scores (e.g. PI3K–Akt pathway, Hippo–Merlin pathway). In line with the above, APNet’s predictive drivers were more clearly associated with known clinical markers of severity (e.g. LDH, CRP) compared to the benchmarking prioritized drivers, thereby highlighting its capacity to better decipher the intricate immunopathology of severe COVID-19 involving multiple-organ failure and exacerbated inflammation across tissues (Ahern *et al.* 2022, Jamison *et al.* 2022).

Despite the valuable advantages of APNet, this study holds the limitation of focusing on a short number of omic datasets, on a particular and well-studied disease, i.e. severe COVID-19. However, APNet can be disease agnostic; thus, it should be implemented in datasets obtained from several diseases, especially rare ones, in the future. In terms of data, using the activity transformations as a gateway, APNet could also be trained on plasma proteomics, RNA-seq or scRNA-seq and then tested on spatial transcriptomics, which would allow for a more spatially aware approach regarding multi-omic dynamics [e.g. Mothes *et al.* (2023) for COVID-19 lung spatial transcriptomics]. Another limitation of this study is the use of Enrichr KG to construct biological priors through one-hot encoding with joint differentially active drivers across studies. Alternative experimentation with priors based on clusters from the SJARACNe networks or pathways after GSEA (based on activity) could lead to more fine-tuned models with increased accuracy and better explainability.

In the future, we would also like to explore switching PASNet with more versatile DL models for clinical predictions. Considering the emergence of novel models, like Cox-PASNet (Hao *et al.* 2019) that incorporate clinical along with biological priors, or sparse interpretable autoencoders (AutoSurv) (Jiang *et al.* 2024), APNet could serve as a modular framework to ‘mix and match’ several DL architectures with activity-transformed omic datasets to create bespoke pipelines. Lastly, another limitation of this work is the script-based logic of APNet as it may prove suboptimal for seamless operations across cloud-based or High Performance Computing (HPC) infrastructures. A potential assembly of relevant NextFlow pipelines could be a promising avenue for improving APNet’s usability and scalability.

Overall, APNet is a robust DL framework that can facilitate the extraction of intricate biological insights from complex biological data along with predictions on clinical outcomes and evaluation of mechanistic hypotheses. In an optimized version of our tool, we aim to escalate the scalability to other multi-factorial disease-omic datasets (such as cancer and neurodegenerative diseases) and explore its potential across various bulk, single-cell, and spatial multi-omic experiments. In vitro/in vivo validations of the predicted indications will strengthen the credibility of our framework and represent the actual benchmark standard of APNet.

## Supplementary Material

btaf063_Supplementary_Data

## Data Availability

APNet R and Python scripts and the code to re-create the figures of this manuscript can be found at https://github.com/BiodataAnalysisGroup/APNet. The datasets used in this study can be accessed in the following links: MGH Olink proteomics: https://info.olink.com/mgh-covid-study-overview-page?hsCtaTracking=fff99a2a-81c1-4e4a-a70d-6922d26503b4 Mayo Olink Proteomics: https://www.thelancet.com/journals/landig/article/PIIS2589-7500(22)00112-1/fulltext#supplementaryMaterial Stanford Olink proteomics: https://datadryad.org/stash/dataset/doi:10.5061/dryad.9cnp5hqmn Single-cell MGH Villani group and Blish: https://www.covid19cellatlas.org/index.patient.html All of the above data are also included in our Zenodo link: (Past version: https://doi.org/10.5281/zenodo.1043883010.5281/zenodo.10438830, New version: https://doi.org/10.5281/zenodo.1468052010.5281/zenodo.14680520).
