# Supplementary material for: APNet, an explainable sparse deep learning model to discover differentially active drivers of severe COVID-19
Source: Bioinformatics. 2025 Feb 8;41(3):btaf063. doi: 10.1093/bioinformatics/btaf063 (PMC11897427; doi:10.1093/bioinformatics/btaf063)
Supplement: btaf063_Supplementary_Data [file btaf063_supplementary_data.zip › APNet_Supplementary_Material.pdf]

**Title**: APNet, an explainable sparse deep learning model to discover differentially active drivers of severe COVID-19

**Supplementary Material (Figures and Tables)**

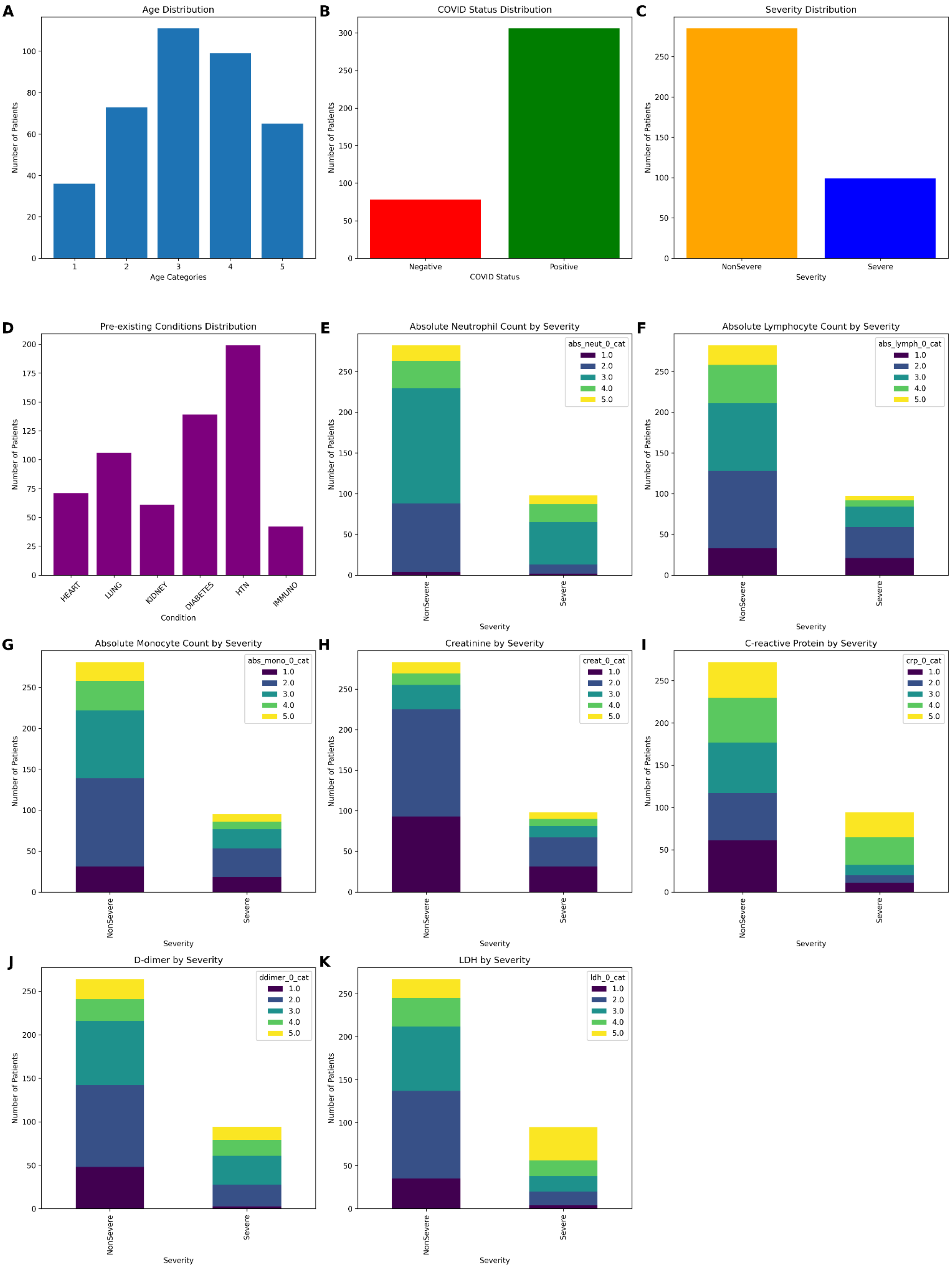
A
Age Distribution
Number of Patients
Age Categories
1
2
3
4
5
B
COVID Status Distribution
Number of Patients
COVID Status
Negative
Positive
C
Severity Distribution
Number of Patients
Severity
NonSevere
Severe
D
Pre-existing Conditions Distribution
Number of Patients
Condition
HEART
LUNG
KIDNEY
DIABETES
HTN
IMMUNO
E
Absolute Neutrophil Count by Severity
abs_neut_0_cat
F
Absolute Lymphocyte Count by Severity
abs_lymph_0_cat
G
Absolute Monocyte Count by Severity
abs_mono_0_cat
H
Creatinine by Severity
creat_0_cat
I
C-reactive Protein by Severity
crp_0_cat
J
D-dimer by Severity
ddimer_0_cat
K
LDH by Severity
ldh_0_cat
1.0
2.0
3.0
4.0
5.0
Number of Patients
Severity
NonSevere
Severe

**Supplementary Figure 1. Demographic and clinical covariates for the bulk Olink MGH patient cohort.** The covariates are described with barplots (A-D) and stacked barplots (G-K)**. (A)** Distribution of patients across five age categories, with the highest number in the third category. **(B)** Distribution of patients by COVID-19 status, showing a majority that is positive. **(C)** Distribution of patients by disease severity, with non-severe cases outnumbering severe cases. **(D)** Distribution of pre-existing conditions, with diabetes and hypertension being most common. **(E)** Absolute neutrophil count by severity, with increased numbers in severe cases. **(F)** Absolute lymphocyte count by severity, showing a marked decrease in severe cases. **(G)** Absolute monocyte count by severity. **(H)** Creatinine levels by severity, with elevated levels in severe cases. **(I)** C-reactive protein levels by severity, higher in severe cases. **(J)** D-dimer levels by severity, showing increased levels in severe cases. **(K)** LDH levels by severity, with higher levels in severe cases.

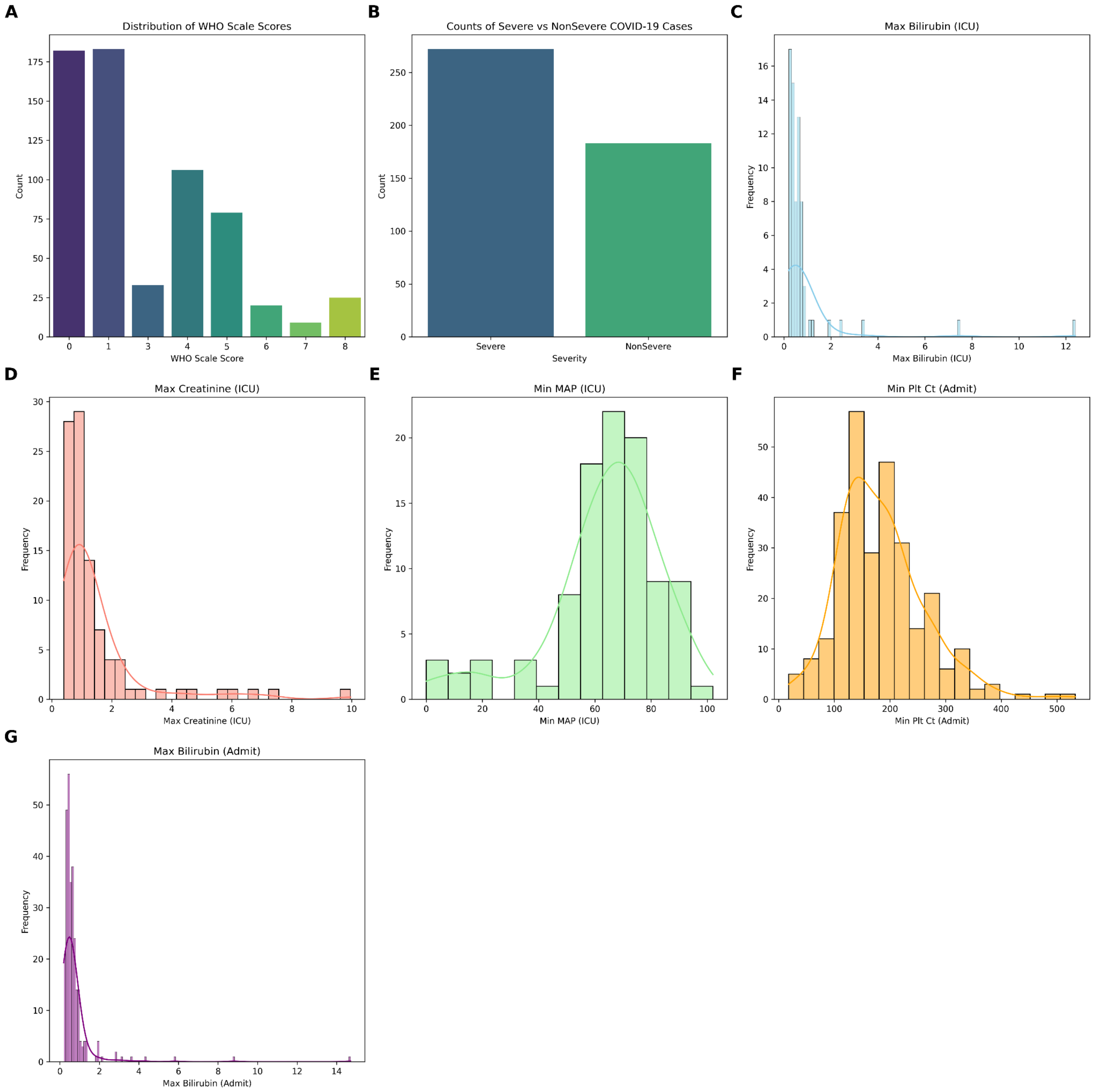


**Supplementary Figure 2. Demographic and clinical covariates for the bulk Olink Mayo patient cohort.** Barplots (A-B) and histograms (C-G) are used to depict the covariates. **(A)** Distribution of WHO scale scores among patients, with the highest count at score 1. **(B)** Counts of severe versus non-severe COVID-19 cases, with severe cases being more abundant. **(C)** Max bilirubin levels in ICU, showing a right-skewed distribution. **(D)** Max creatinine levels in ICU, with a right-skewed distribution. **(E)** Minimum MAP (mean arterial pressure) in ICU, showing a normal distribution. **(F)** Minimum platelet count at admission, with a normal distribution. **(G)** Max bilirubin levels at admission, exhibiting a right-skewed distribution.

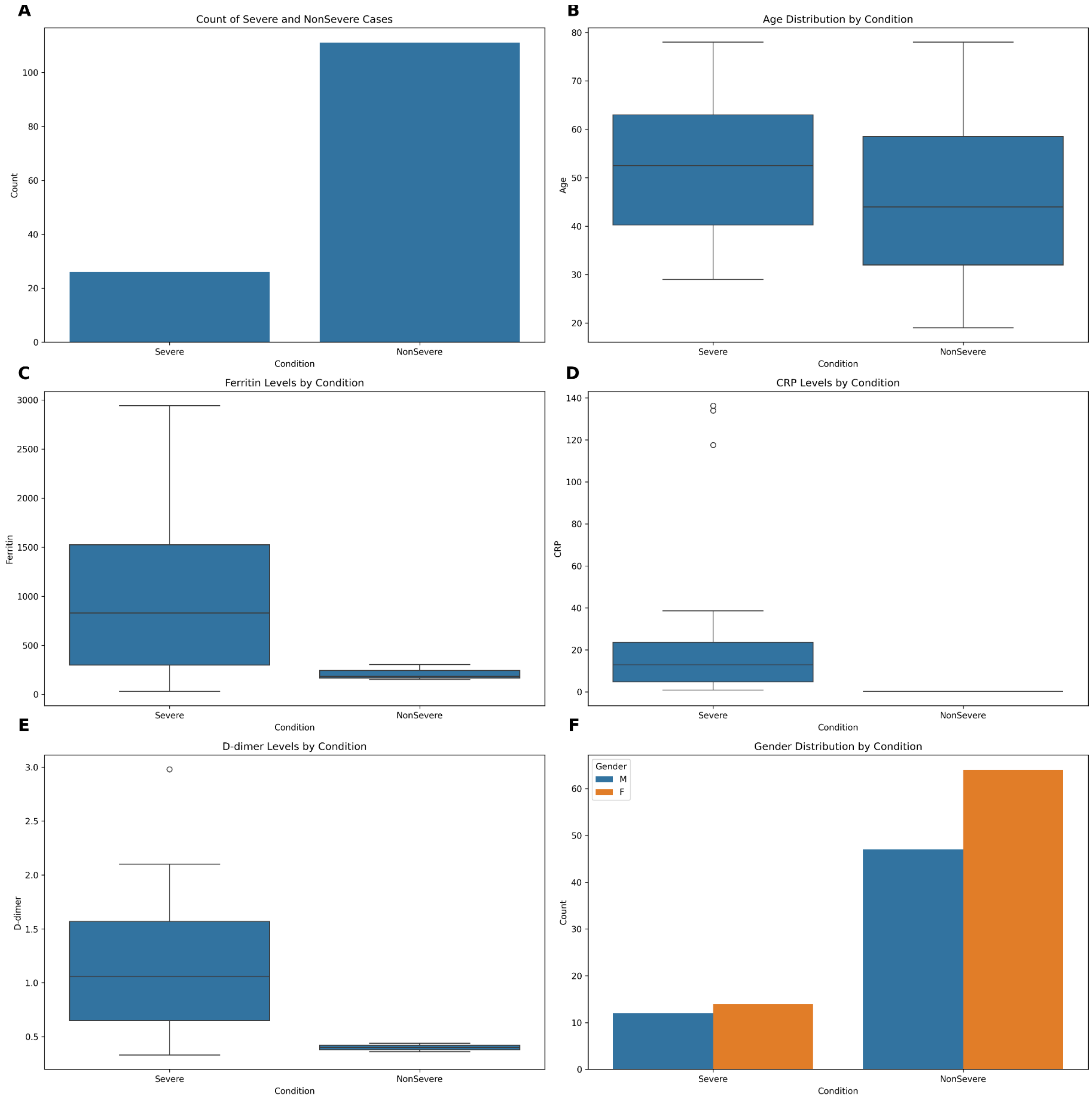


**Supplementary Figure 3. Demographic and clinical covariates for the bulk Olink Stanford patient cohort.** Covariates are described in barplots. **(A)** Count of severe versus non-severe cases, with non-severe cases being more frequent. **(B)** Age distribution by condition, showing similar medians but wider ranges in severe cases. **(C)** Ferritin levels by condition, with higher levels in non-severe cases. **(D)** CRP levels by condition, showing higher levels and more variability in severe cases. **(E)** D-dimer levels by condition, with elevated levels in severe cases. **(F)** Gender distribution by condition, showing counts of male and female patients in severe and non-severe categories, with more females in non-severe cases.

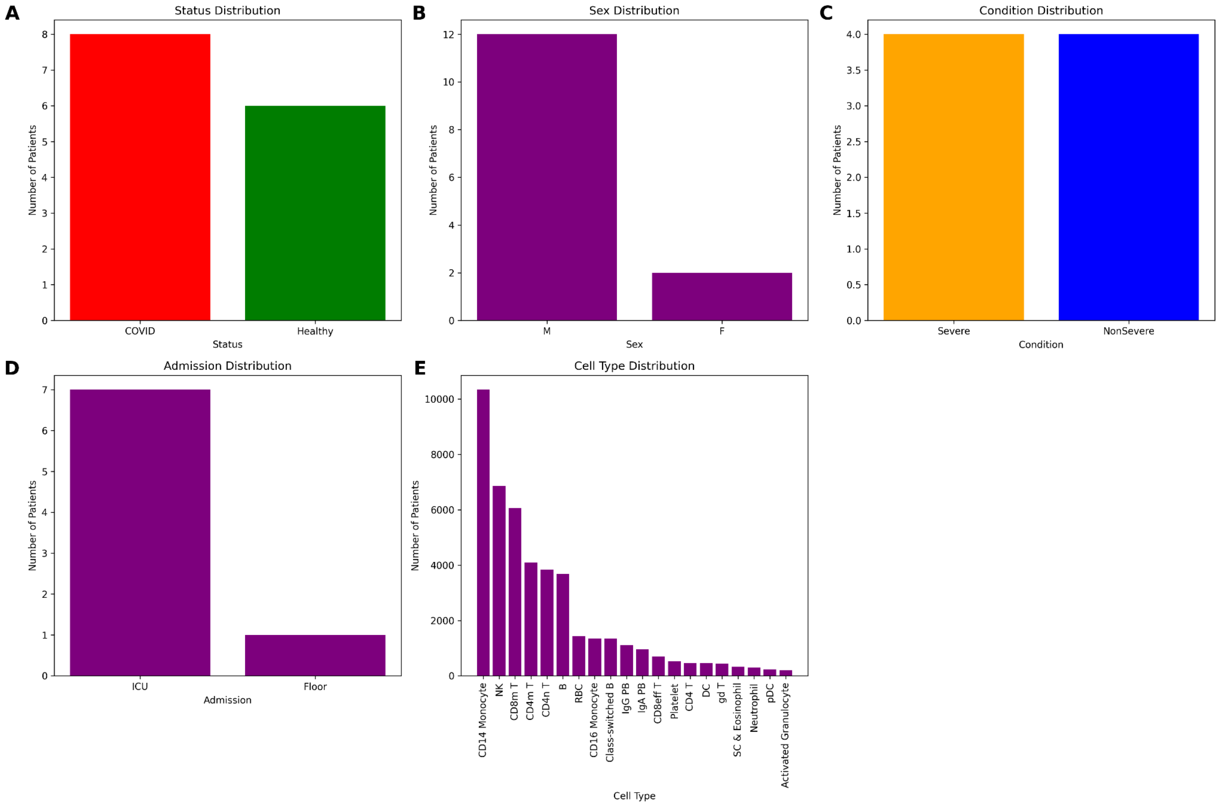


**Supplementary Figure 4. Demographic and clinical covariates for the Blish scRNAseq patient cohort.** Covariates are described in barplots. **(A)** Count of Covid versus Healthy samples, with COVID cases being more frequent, across all Blish dataset. **(B)** Gender distribution across samples from datasets. **(C)** Condition Distribution in COVID patients, by severe versus non-severe, showing that the number of samples of severe and nonsevere. **(D)** Illustrates the Admission Levels of COVID-19 patients, categorizing their care requirements from mild to critical severity. **(E)** Cell type Distribution across examined scRNAseq dataset.

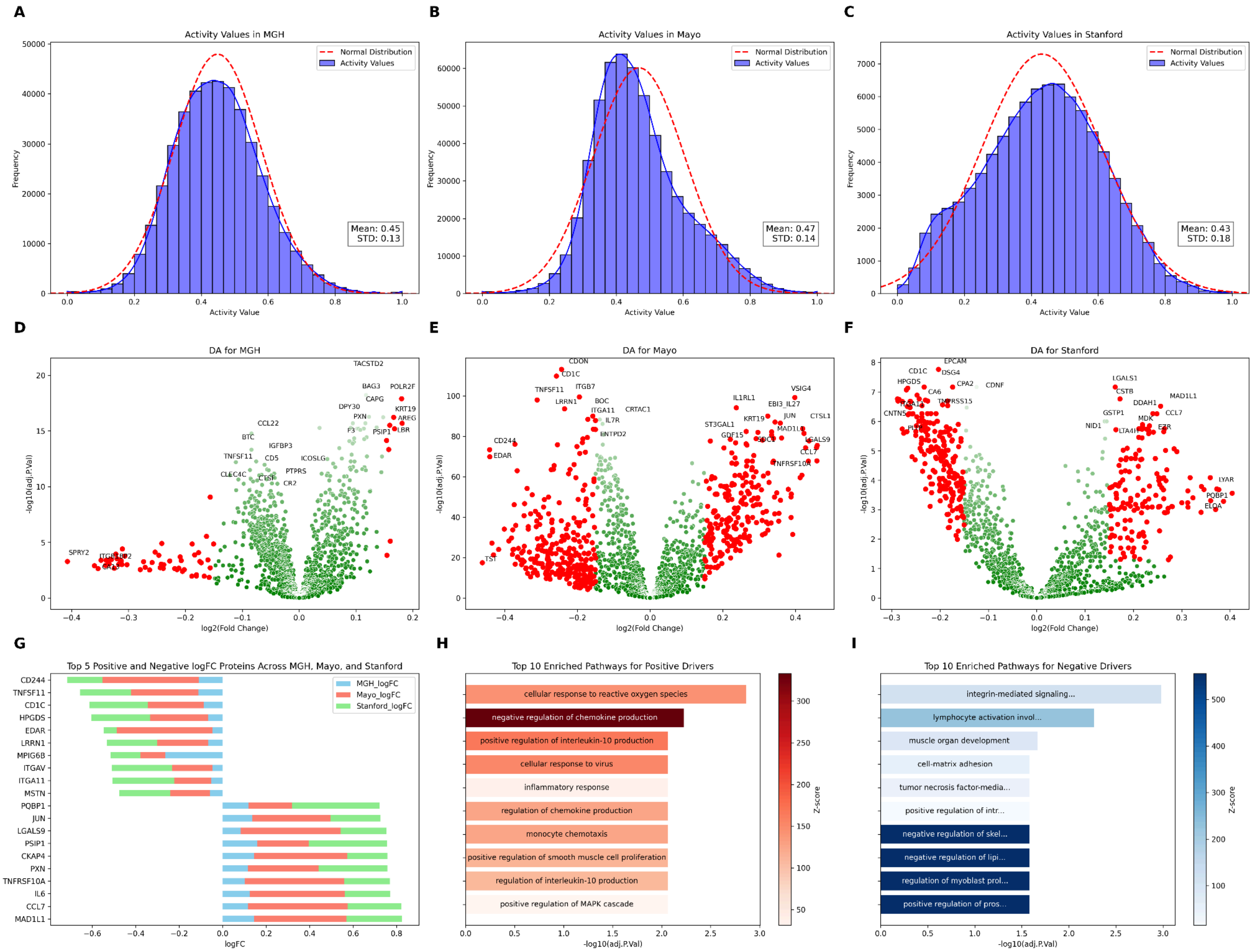


**Supplementary Figure 5. The effect of activity transformations on the bulk plasma proteomics in terms of data distribution, differential change in severe COVID-19 and underlying biology. (A-C)** Distributions of activity values per Olink dataset for **(A)** MGH, **(B)** Mayo, and **(C)** Stanford. Mean and standard deviation are depicted. **(D-F)** Volcano plots that show the most critical drivers from differential activity analysis per dataset. **(G)** Barplot showing cumulating log fold-changes (logFCs) across the three studies for highly hyper-active and markedly hypo-active drivers. Light blue denotes the contribution of MGH dataset, light red that of Mayo dataset and light green the contribution of Stanford dataset. **(H-I)** Following the results in (G), barplots depicting the top 10 pathway enrichment

results of the top 10 hyper-active drivers from (H) and 10 hypo-active ones (I), ordered by -log(adj.p.value) and coloured by their z-score.

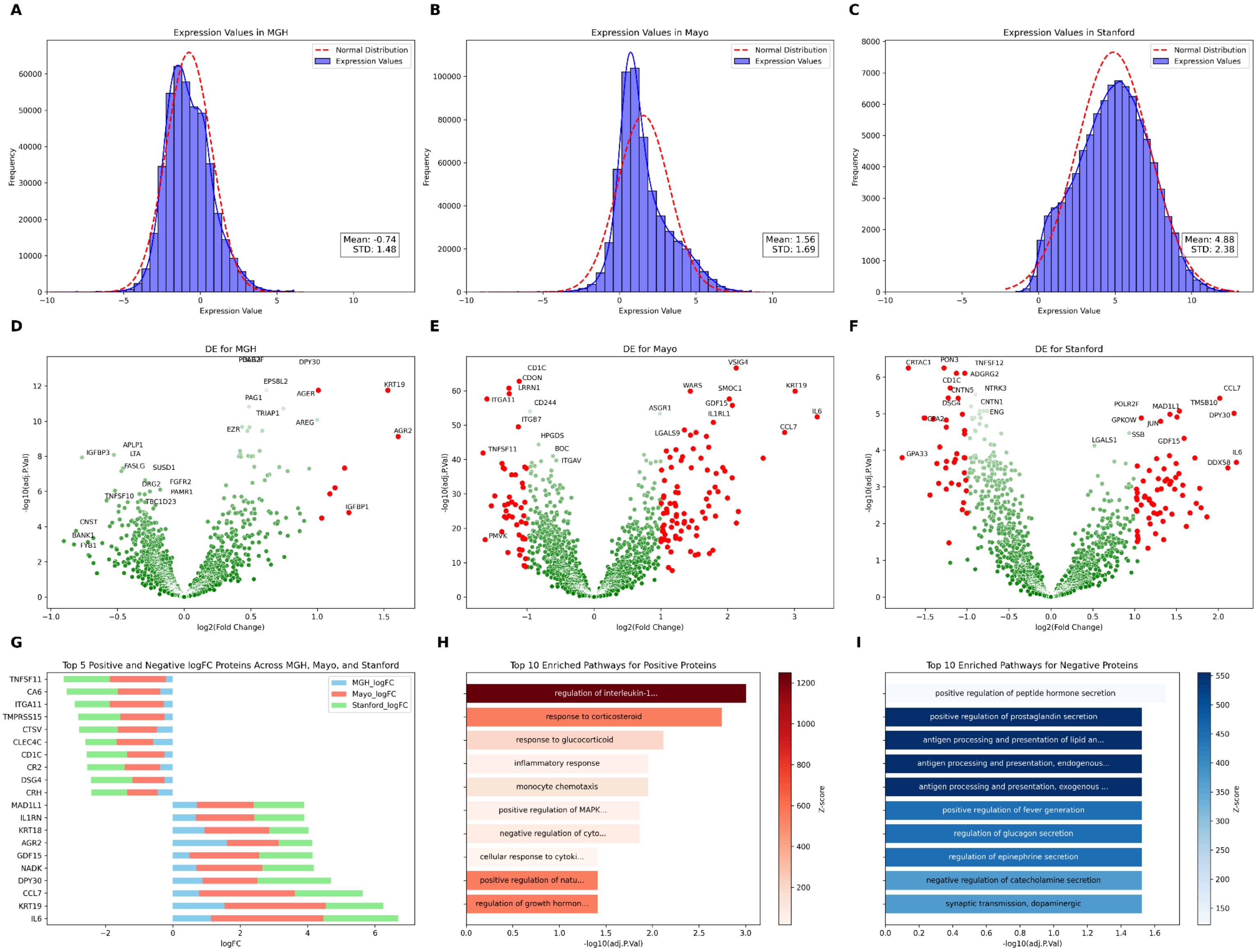


**Supplementary Figure 6. A more typical analysis on bulk plasma proteomics based on their normalised expression values, in terms of data distribution, differential change in severe COVID-19 and underlying biology. (A-C)** Distributions of normalised expression values per Olink dataset for **(A)** MGH, **(B)** Mayo, and **(C)** Stanford. Mean and standard deviation are depicted. **(D-F)** Volcano plots that show the most critical drivers from differential expression analysis per dataset. **(G)** Barplot showing cumulating log fold-changes (logFCs) across the three studies for highly hyper-active and markedly hypo-active drivers. Light blue denotes the contribution of MGH dataset, light red that of Mayo dataset and light green the contribution of Stanford dataset. **(H-I)** Following the results in (G), barplots depicting the top 10 pathway enrichment results of the top 10 hyper-active drivers from (H) and 10 hypo-active ones (I), ordered by -log(adj.p.value) and coloured by their z-score.

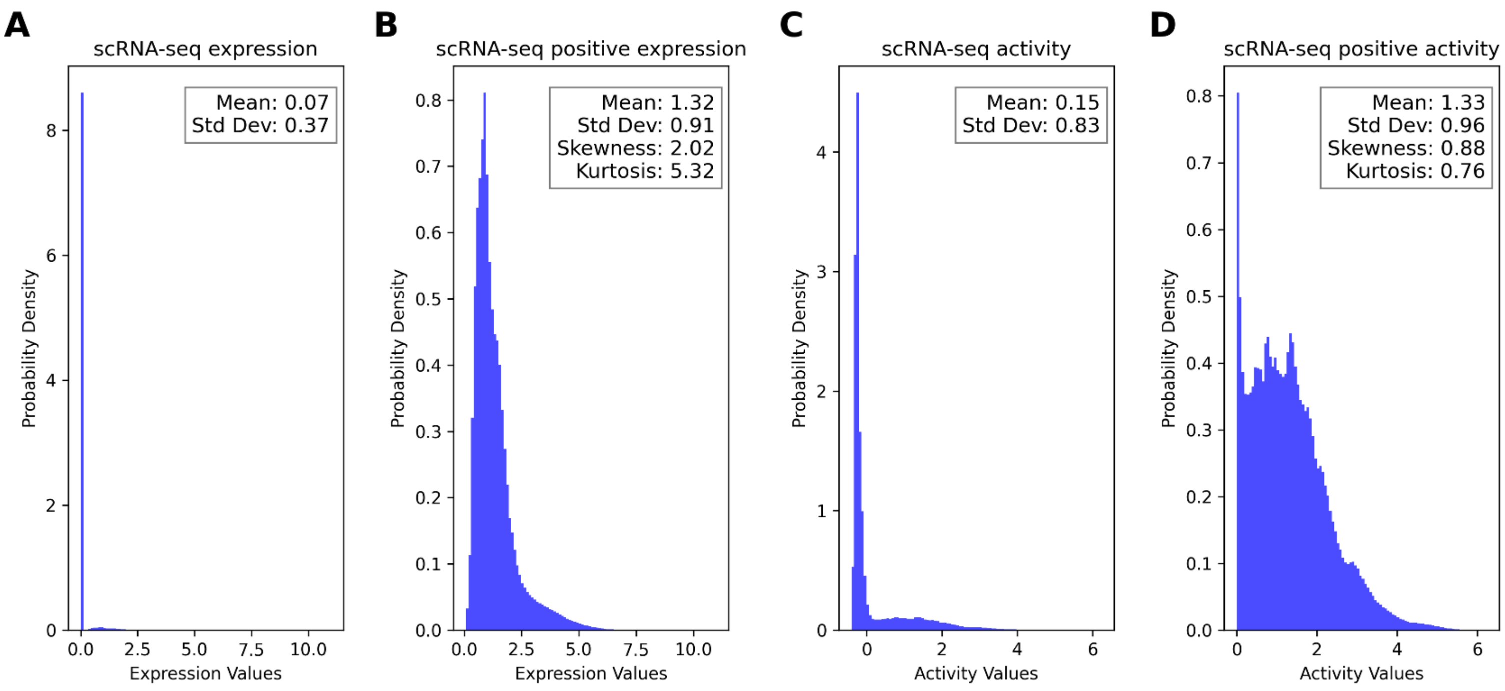


**Supplementary Figure 7. Representation of distributions of scRNA-seq MGH data before and after activity transformations. (A-B)** Distribution of log-normalised expression values (A all and B only the non-zero, positive ones) from the PBMC scRNA-seq COVID-19 data. **(C-D)** Same as (A-B) but after scMINER pre-processing for activity values. Distribution statistics are depicted in each panel.

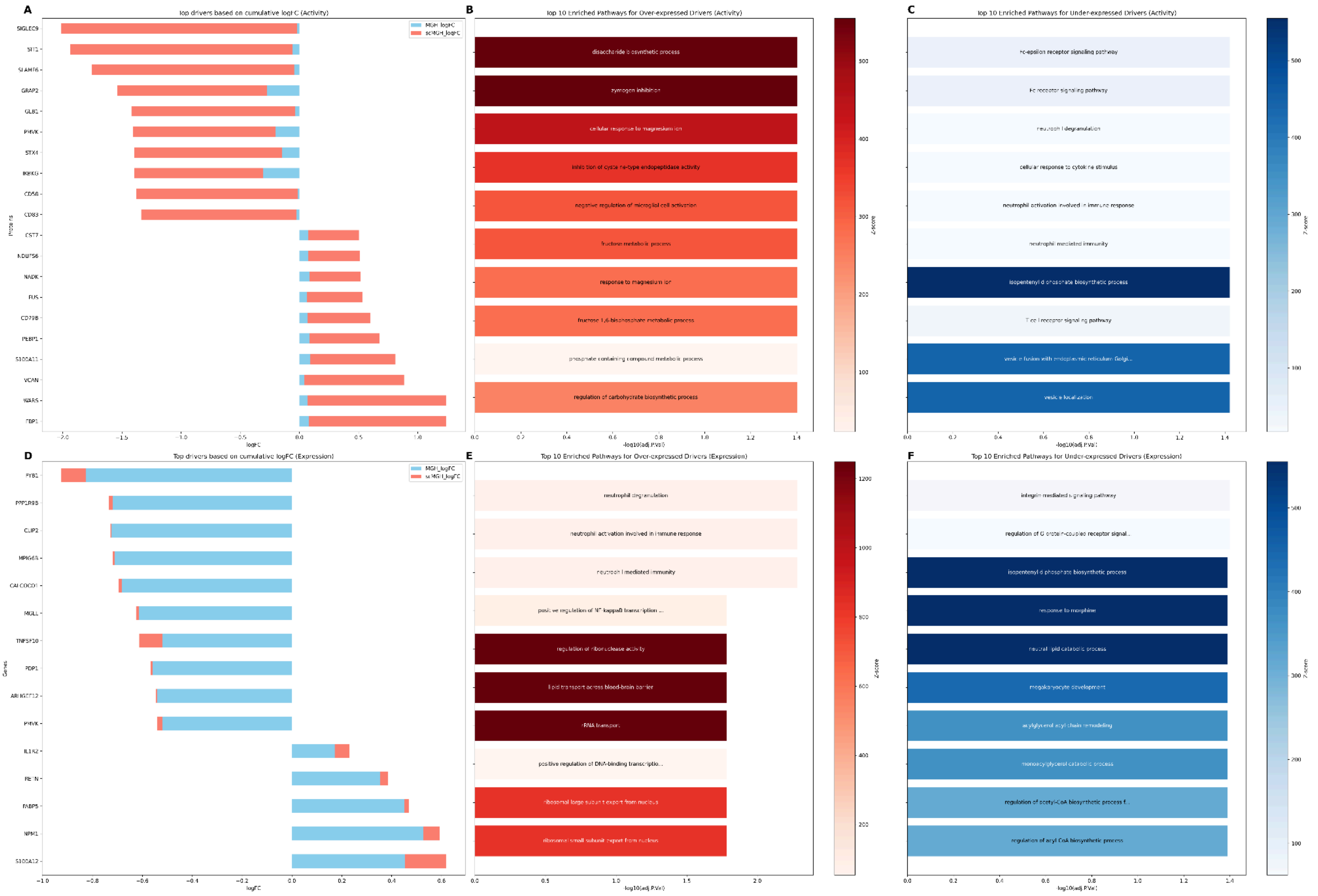


**Supplementary Figure 8. Differential activity analysis prioritises different drivers of COVID-19 severity than differential expression analysis (A)** Stacked Bar plot illustrating the cumulative log

fold-changes (logFCs) from differential activity analyses of Olink MGH proteomics and MGH single cells RNAseq datasets for highly hyperactive and significantly hypoactive drivers. The light blue bars represent the contribution from the MGH dataset, while the light red bars indicate the scMGH dataset. **(B-C)** Based on the findings in (A), bar plots displaying the top 10 enriched pathways for the top 10 hyperactive drivers (B) and the top 10 hypoactive drivers (C), ranked by -log(adj.p.value) and color-coded according to their z-scores. **(D)** Bar plot illustrating the cumulative log fold-changes (logFCs) from differential expression analyses of Olink MGH proteomics and MGH single-cell RNAseq datasets for highly overexpressed and significantly underexpressed proteins/genes. The light blue bars represent the contribution from the MGH dataset, while the light red bars indicate the scMGH dataset. **(E-F)** Based on the findings in (D), bar plots displaying the top 10 enriched pathways for the top 10 overexpressed proteins (E) and the top 10 underexpressed proteins (F), ranked by -log(adj.p.value) and color-coded according to their z-scores.

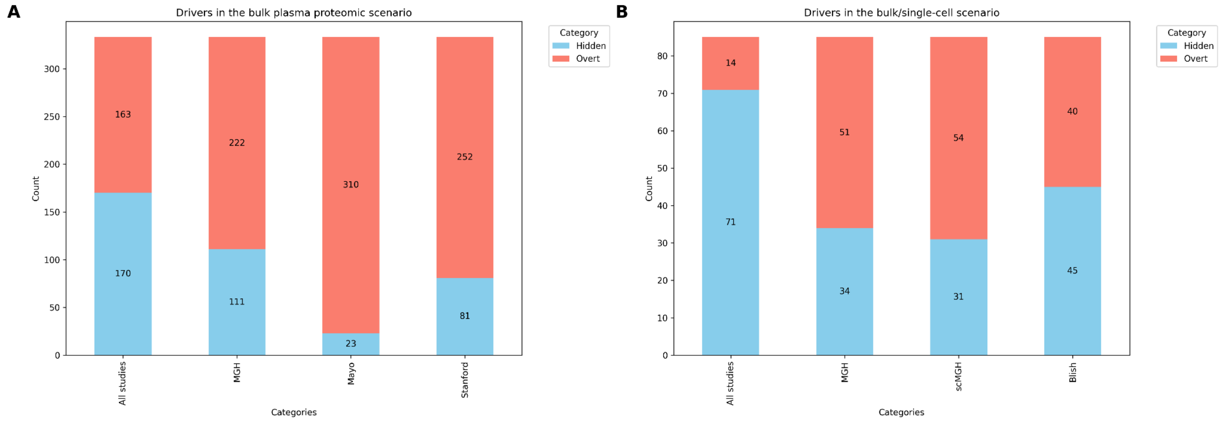


**Supplementary Figure 9. An overview of overt and hidden drivers across 3 bulk Olink proteomic and scRNAseq datasets. (A)** Stacked Bar plots showing the number of types (Overt - Hidden) severity drivers across Olink proteomics datasets. **(B)** Same as (A) but for the bulk/single-cell MGH and Blish scenario.

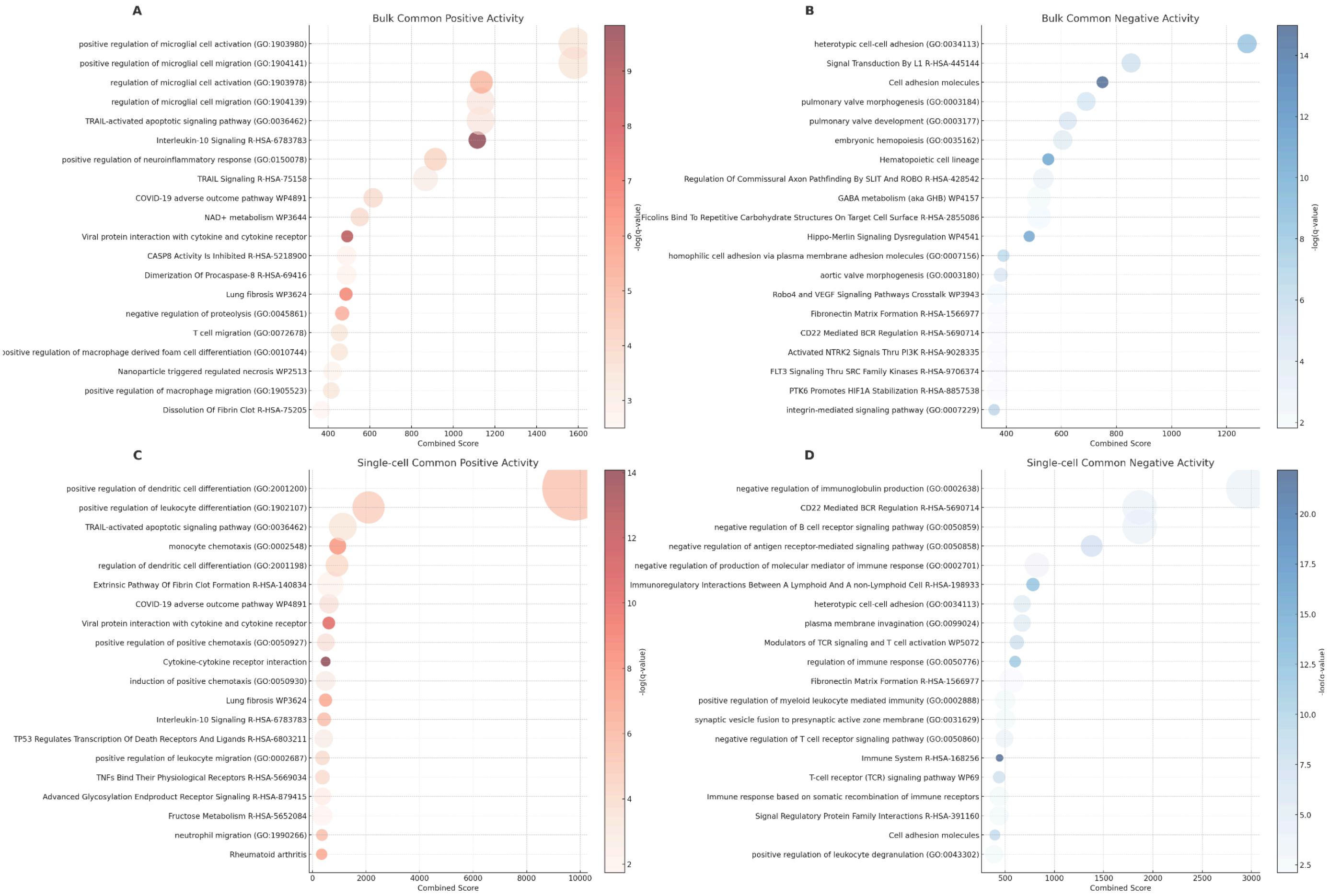


**Supplementary Figure 10. Pathway enrichment for DAPs and DAPs/DAGs in bulk and single-cell scenarios. (A-B)** Bubble Plots showing top pathways from the assembly of deep learning biological priors as constructed from Enrichr Knowledge Graph (KG) for the joint differentially hyper-active (A) and hypo-active drivers (B) of COVID-19 severity from the MGH-Mayo and MGH-Stanford scenarios. **(C-D)** Same as (A-B) but for the joint hyper-active (C) and hypo-active drivers from the bulk proteomic/scRNA-seq scenario within the MGH study. The Enrichr KG has provided pathways from WikiPathway_2021_Human, Reactome_2022, GO:BP 2021 and KEGG_2021_human.

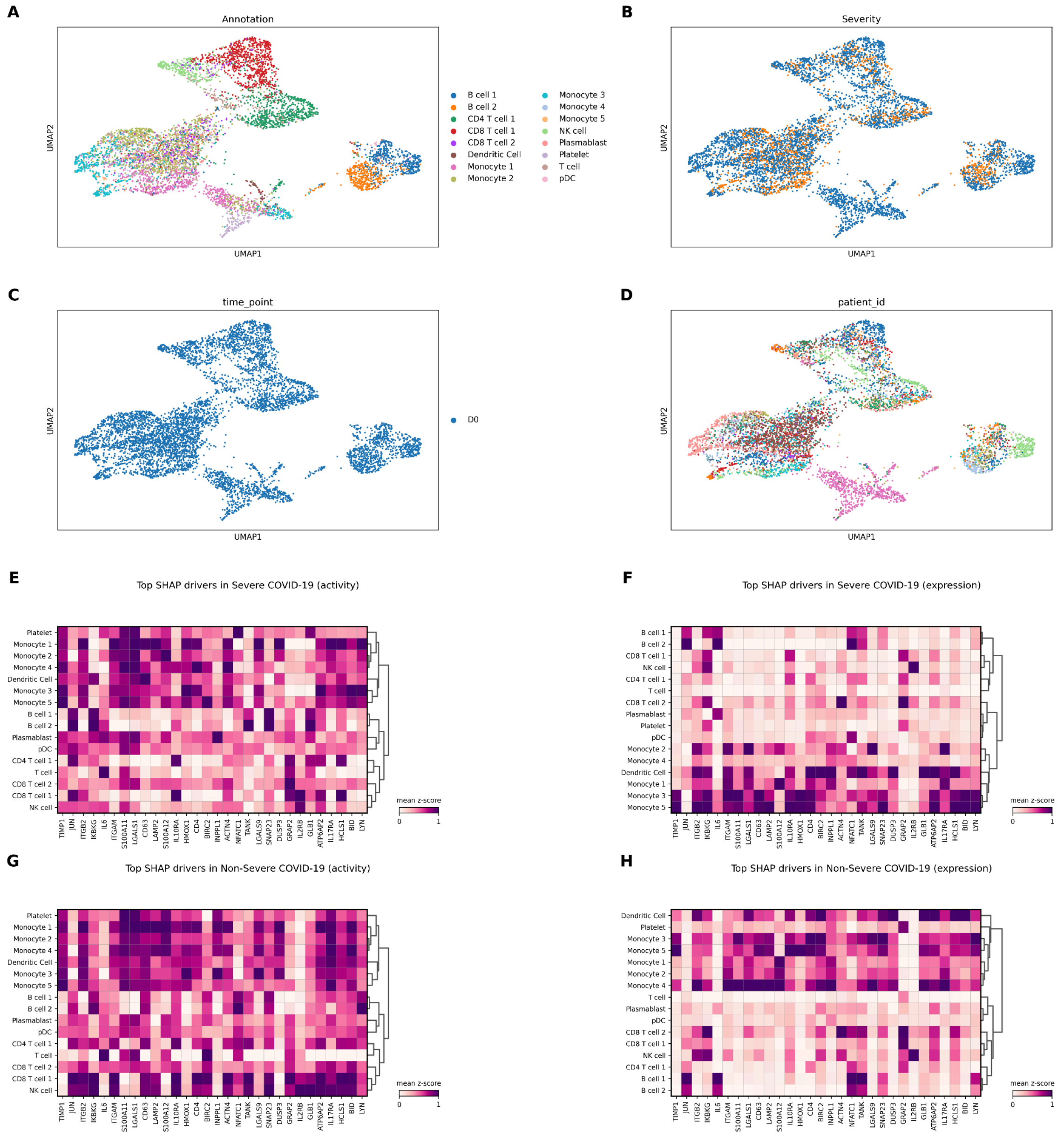


**Supplementary Figure 11: Representation of MGH single-cell RNAseq dataset post-APNet analysis. (A-D)** UMAP plots of the PBMC COVID-19 scRNA-seq dataset indicating the annotated cell type of dataset (A), COVID-19 severity (B), the timepoint of analysis (C) and the number of patients (D). **(E-F)** Matrix plots showing the top most predictive drivers from the APNet deployment on the bulk proteomic/scRNA-seq MGH scenario in severe COVID-19 cases with activity values (E) and expression values (F). **(G-H)** Same as (E-F) but for the non-severe COVID-19 cases. Visualisations were attained using Scanpy package in Python.

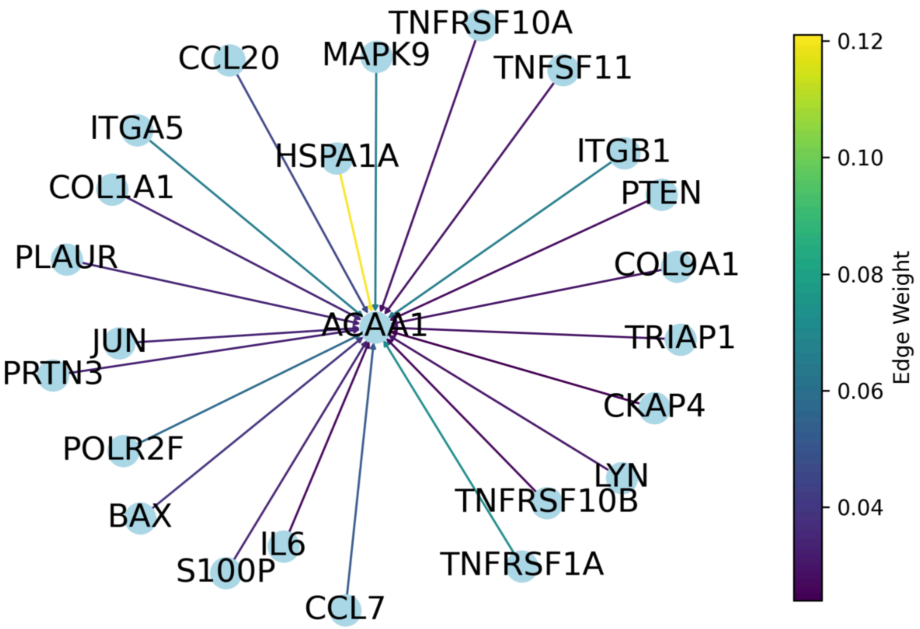


**Sup Figure 12. Representation of the ACAA1 regulon derived from the top SHAP genes.** This graph visualizes the regulatory interactions of ACAA1, identified as a significant driver, based on GENIE3 algorithm analysis. Edges are weighted by the GENIE3 output, with a color gradient from blue (low weight) to yellow (high weight), representing the strength of the interactions.

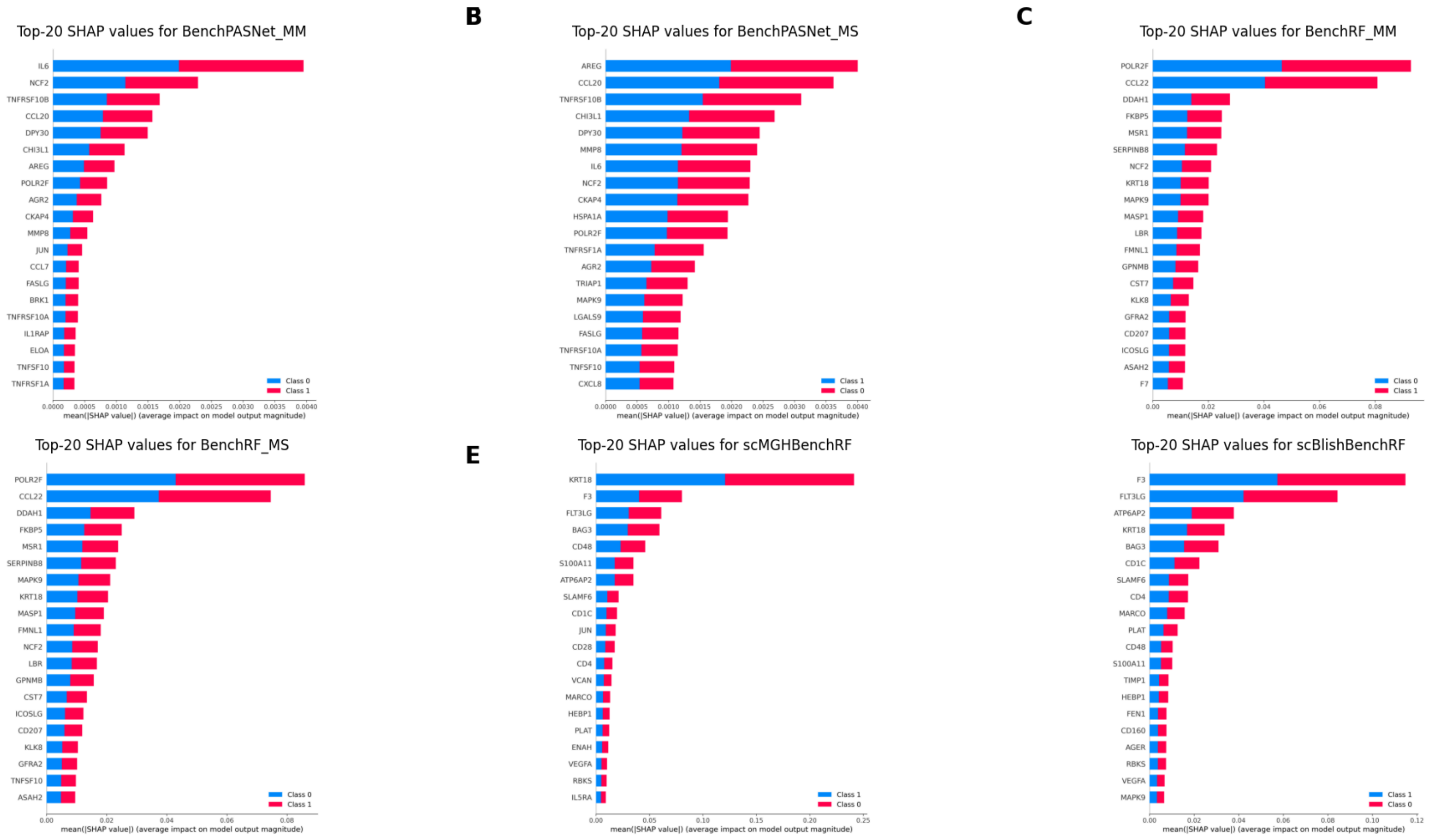


**Supplementary Figure 13.** Barplots showing top SHAP drivers from benchmark models (PASNet with expression values, Random Forest/RF) competing with APNet models.

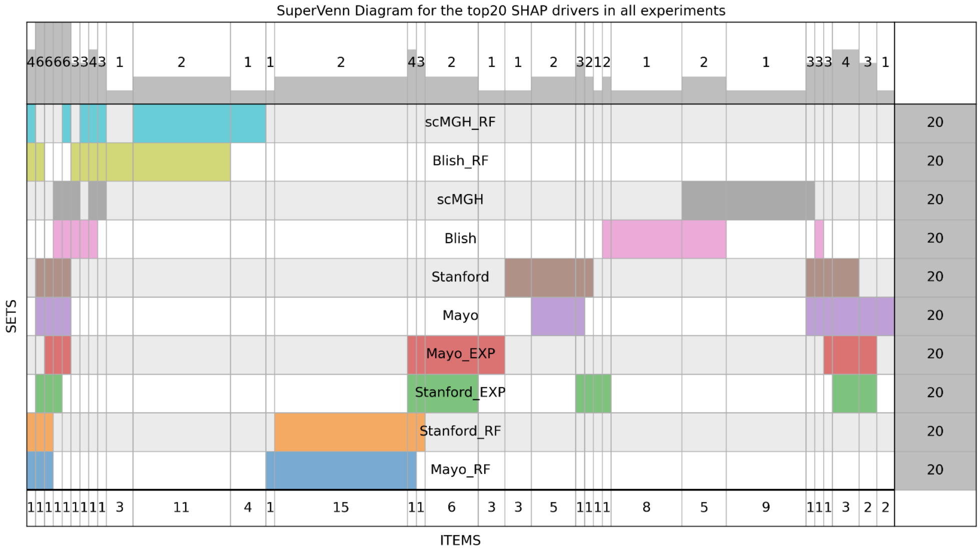


**Supplementary Figure 14.** SuperVenn plot showing commonalities and differences among the top-20 most predictive drivers (based on SHAP values) from APNet deployment and benchmarking models across all scenarios.

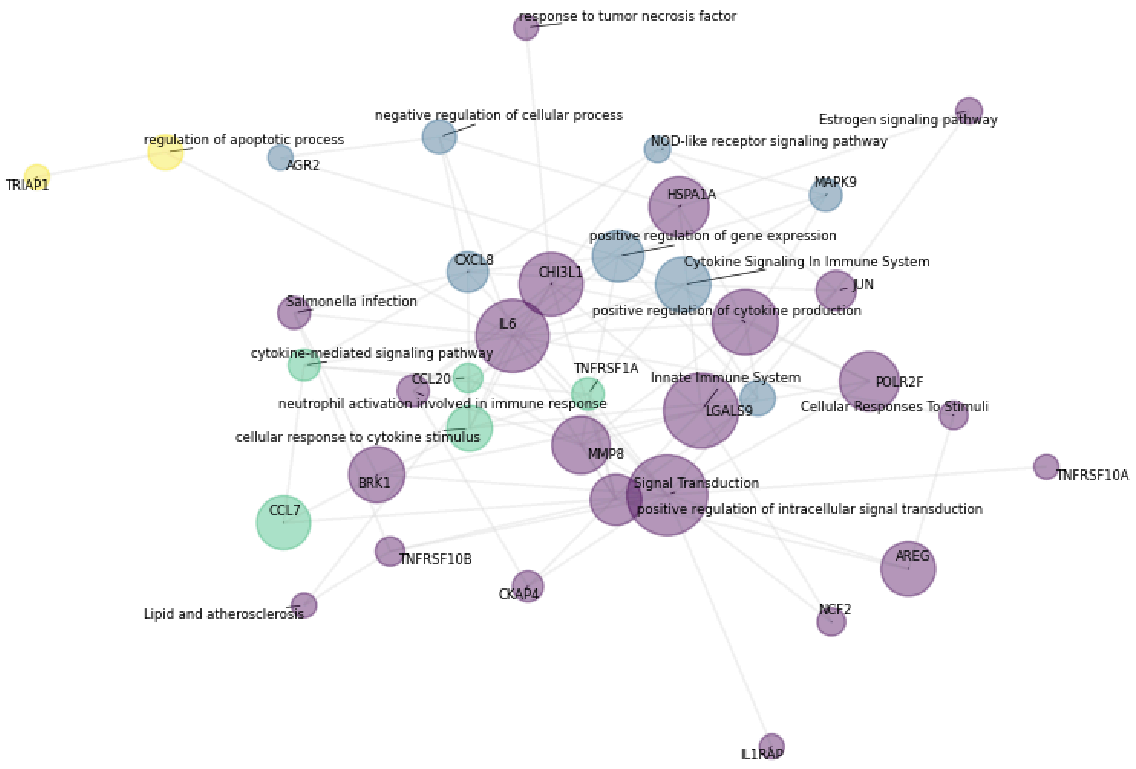


**Supplementary Figure 15. (A-B)** Driver-pathway bipartite graphs from BenchPASNet models after running the original PASNet model on the normalised expression of MGH (training), Mayo (validation/testing) and Stanford (testing). Colour is determined based on network communities, while node size is based on betweenness-centrality.

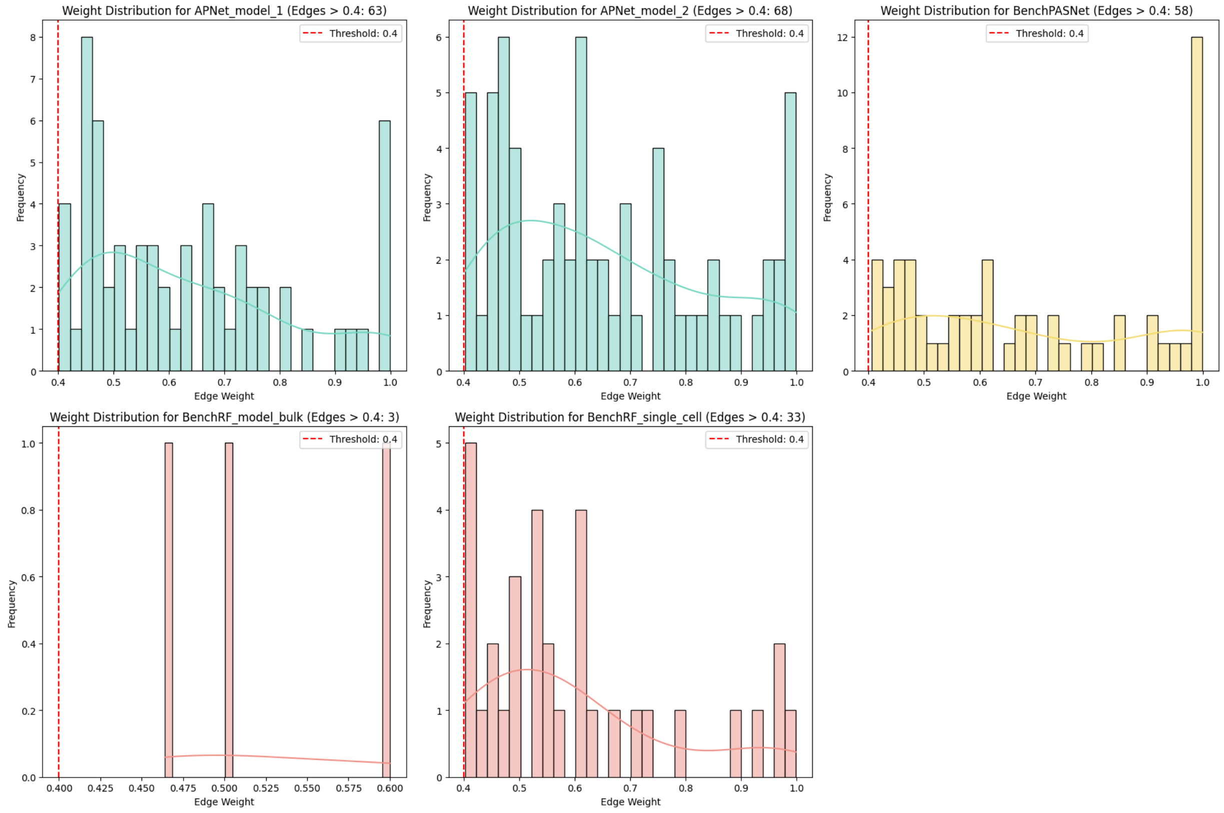


**Supplementary Figure 16.** Histograms showing the frequency of edges in the various STRINGdb PPI networks for the top 20 most predictive drivers across all scenarios (see Figure 7) that have a combined STRINGdb score > 0.4.

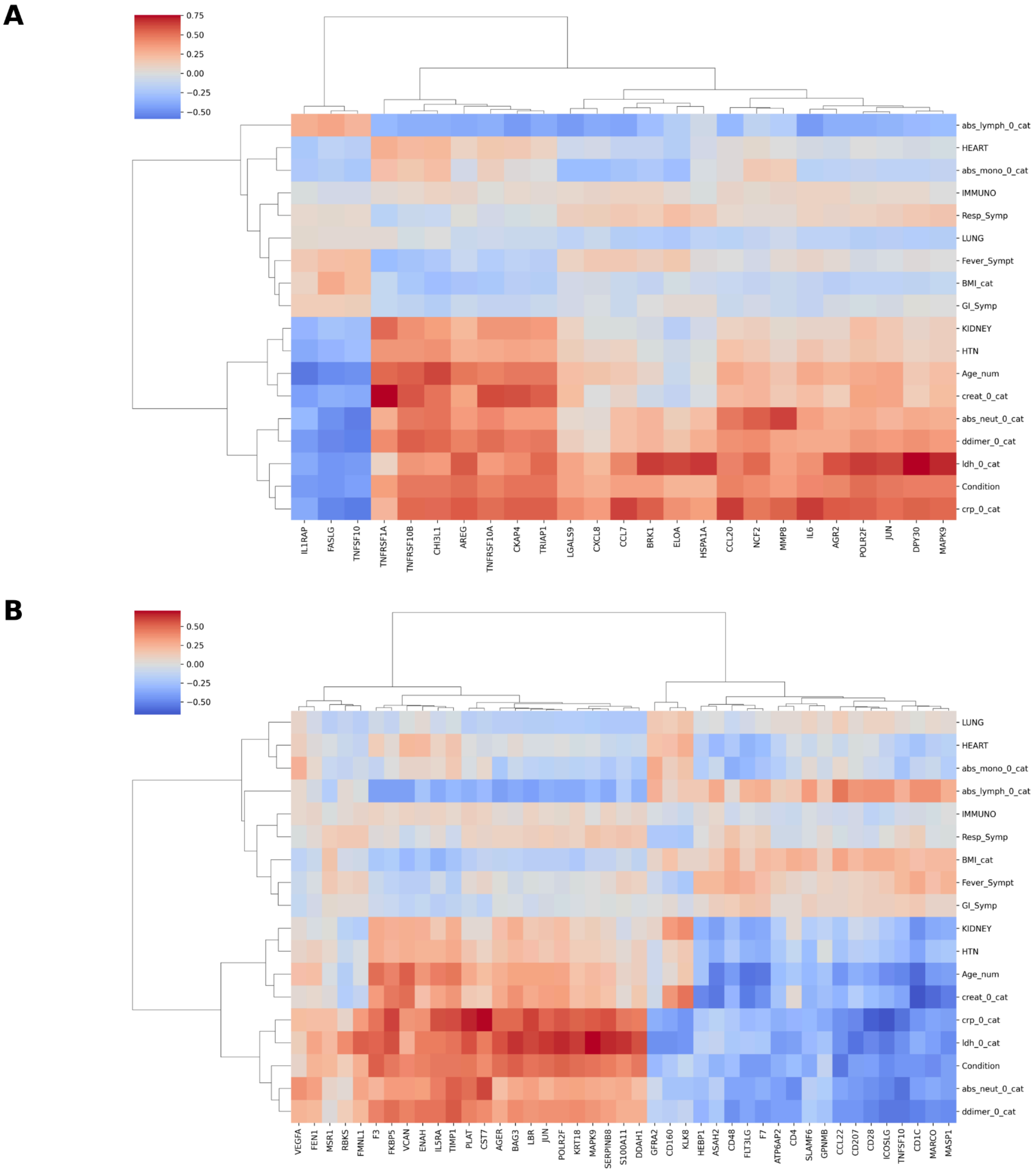


**Supplementary Fugure 17. Pearson correlation heatmap for the top SHAP predictive drivers of Benchmarking approaches and clinical covariates from the MGH dataset. (A)** SHAP values excluded from the PASNet Benchmarking, highlighting their correlation with established clinical markers associated with severe COVID-19. **(B)** SHAP values derived from the Benchmarking using the Random Forest algorithm, illustrating their association with clinical covariates related to severe COVID-19.

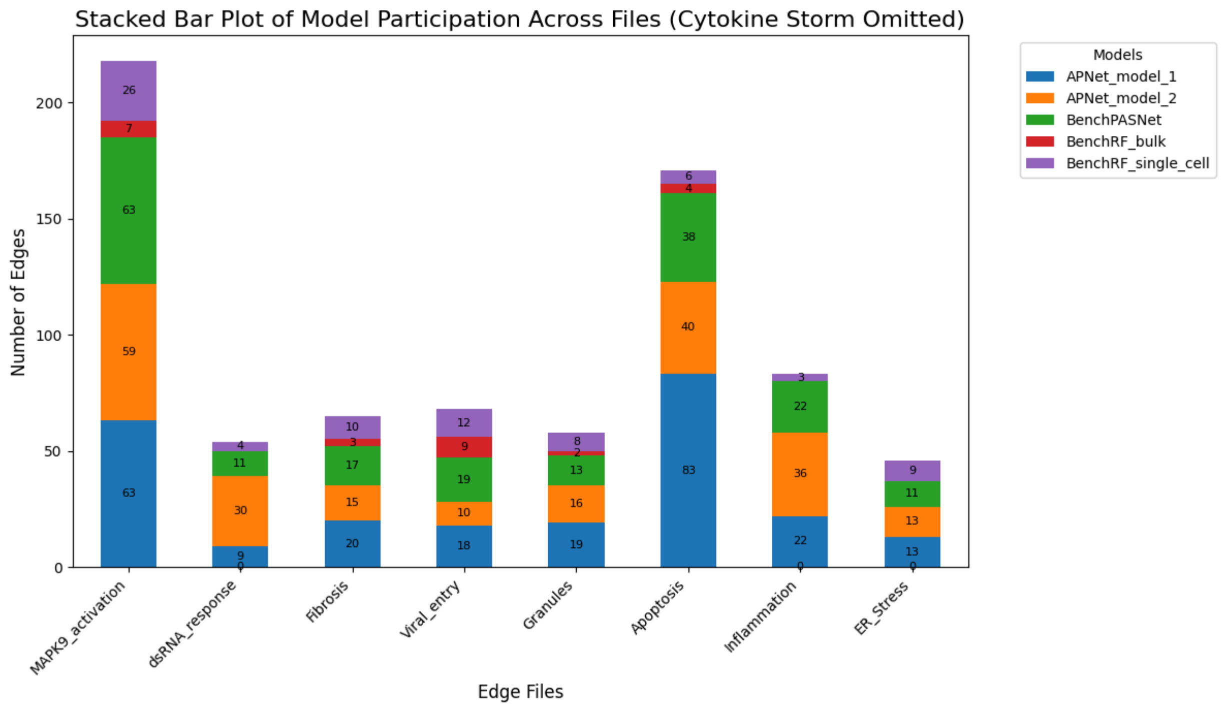


**Supplementary Figure 18.** Stacked Barplots showing the number of edges that the top SHAP proteins from each model participate in the SIGNOR project's curated directed graphs (https://signor.uniroma2.it/covid/).

**Supplementary Table 1. Mean and Standard Deviation of Expression and Activity values across omics datasets.**

| Category | Dataset | Mean | Standard Deviation |
|---|---|---|---|
| Activity Values | MGH | 0.449 | 0.130 |
| | Mayo | 0.468 | 0.137 |
| | Stanford | 0.432 | 0.180 |
| | scMGH** | 0.154 | 0.829 |
| Expression Values | MGH* | -0.740 | 1.483 |
| | Mayo* | 1.56 | 1.693 |
| | Stanford* | 4.885 | 2.3778 |
| | scMGH** | 0.073 | 0.367 |

**: NPX normalised values*
***: non-zero values of the count matrix*

**Supplementary Table 2. Distributions of scRNA-seq Blish data before and after activity transformations**

| Type | Mean | StdDev | Skewness | Kurtosis |
|---|---|---|---|---|
| Activity | 0.07873 | 0.1320 | 3.4636 | 14.13 |
| Positive Activity | 0.0797 | 0.1325 | 3.444 | 13.96 |
| Expression | 0.0194 | 0.0873 | 5.4137 | 38.84 |
| Positive Expression | 0.3611 | 0.1363 | 3.2908 | 18.1528 |

**Supplementary Table 3. Indicative pathways retrieved by APNet across the various scenarios regarding COVID-19 immunopathology**

| Pathways | Citation |
|---|---|
| Immune Signalling | https://doi.org/10.1038/s41577-021-00656-2 |
| VEGFR Signalling | https://doi.org/10.1002/EXP.20210051 |
| Apoptotic Pathways | https://doi.org/10.1038/s41392-023-01580-8 |
| Necrotic Pathways | https://doi.org/10.1016/j.nmni.2020.100738 |
| Neutrophil Activation | https://doi.org/10.1159/000535541 |
| PI3K-Akt Signalling | https://doi.org/10.1016/j.drudis.2021.11.002 |
| Ras Signalling | https://doi.org/10.1007/s00284-023-03304-1 |
| MAPK Signalling | https://doi.org/10.1016/j.biopha.2021.112420 |
| Cytokine Storm | https://doi.org/10.1002/jmv.26232 |
| Oxidative Stress | https://doi.org/10.1134/S0006297920120068 |
| Neuroinflamation | https://doi.org/10.21203/rs.3.rs-1031824/v1 |
| ECM Remodelling | https://doi.org/10.1007/s00011-021-01487-6 |
| Hippo-Merlin Signaling | https://doi.org/10.1101/2022.04.07.487520 |
| Inflammatory Signaling | https://doi.org/10.1159/000535541 |
| Lipid Metabolism | https://doi.org/10.1038/s41598-023-43189-5 |
| Nf-KB Signalling | https://doi.org/10.1038/s41440-023-01460-2 |
| Chemokine Signaling | https://doi.org/10.3389/fimmu.2022.832394 |
| Immune Signalling | https://doi.org/10.1038/s41577-021-00656-2 |
| T-cell Activation | https://doi.org/10.1038/s41577-020-0402-6 |

**Supplementary Table 4.** Unimodality/bimodality statistical metrics for the STRINGdb combined scores as edge weights, deriving from the STRINGdb networks of the top SHAP proteins from the various models.

| Model | Skewness | Kurtosis | Log-Likelihood Ratio | AIC (1 Component) | AIC (2 Components) | BIC (1 Component) | BIC (2 Components) |
|---|---|---|---|---|---|---|---|
| APNet_model_1 | 705 | -634 | 11.062 | -32.99 | -49.12 | -28.7 | -38.4 |
| APNet_model_2 | 471 | -995 | 7.696 | -32.68 | -42.07 | -28.24 | -30.98 |
| BenchPASNet | 272 | -1.453 | 19.883 | -10.38 | -44.15 | -6.26 | -33.85 |
| BenchRF_model_bulk | 469 | -1.5 | 4.788 | -4.66 | -8.23 | -6.46 | -12.74 |
| BenchRF_single_cell | 966 | -162 | 7.253 | -16.65 | -25.16 | -13.66 | -17.67 |
