# Supplementary material for: APNet, an explainable sparse deep learning model to discover differentially active drivers of severe COVID-19
Source: Bioinformatics. 2025 Feb 8;41(3):btaf063. doi: 10.1093/bioinformatics/btaf063 (PMC11897427; doi:10.1093/bioinformatics/btaf063)
Supplement: btaf063_Supplementary_Data [file btaf063_supplementary_data.zip › APNet_Supplementary_Note_Activity_vs_Expression.pdf]

Supplementary Note on the activity concept

The concept of “activity” has been introduced by the authors of NetBID2 (https://doi.org/10.1038/s41467-023-38335-6) and scMINER (https://doi.org/10.1101%2F2023.01.26.523391) as a measure of how potent as a signal driver a molecule of interest is. The potency of a regulatory molecule, such as a transcription factor, lies in its ability to regulate its target molecules, which may not always align with its expression levels in a disease setting. Experimental evidence has shown that some transcription factors can exhibit minimal differential expression that is not statistically significant, yet their actual activity can be strong or weak. This counter-intuitive discrepancy between expression and activity is due to the non-linear dynamics that govern signalling cascades in cell physiology and can be attributed to factors like post-translational modifications, protein-complex stability, and

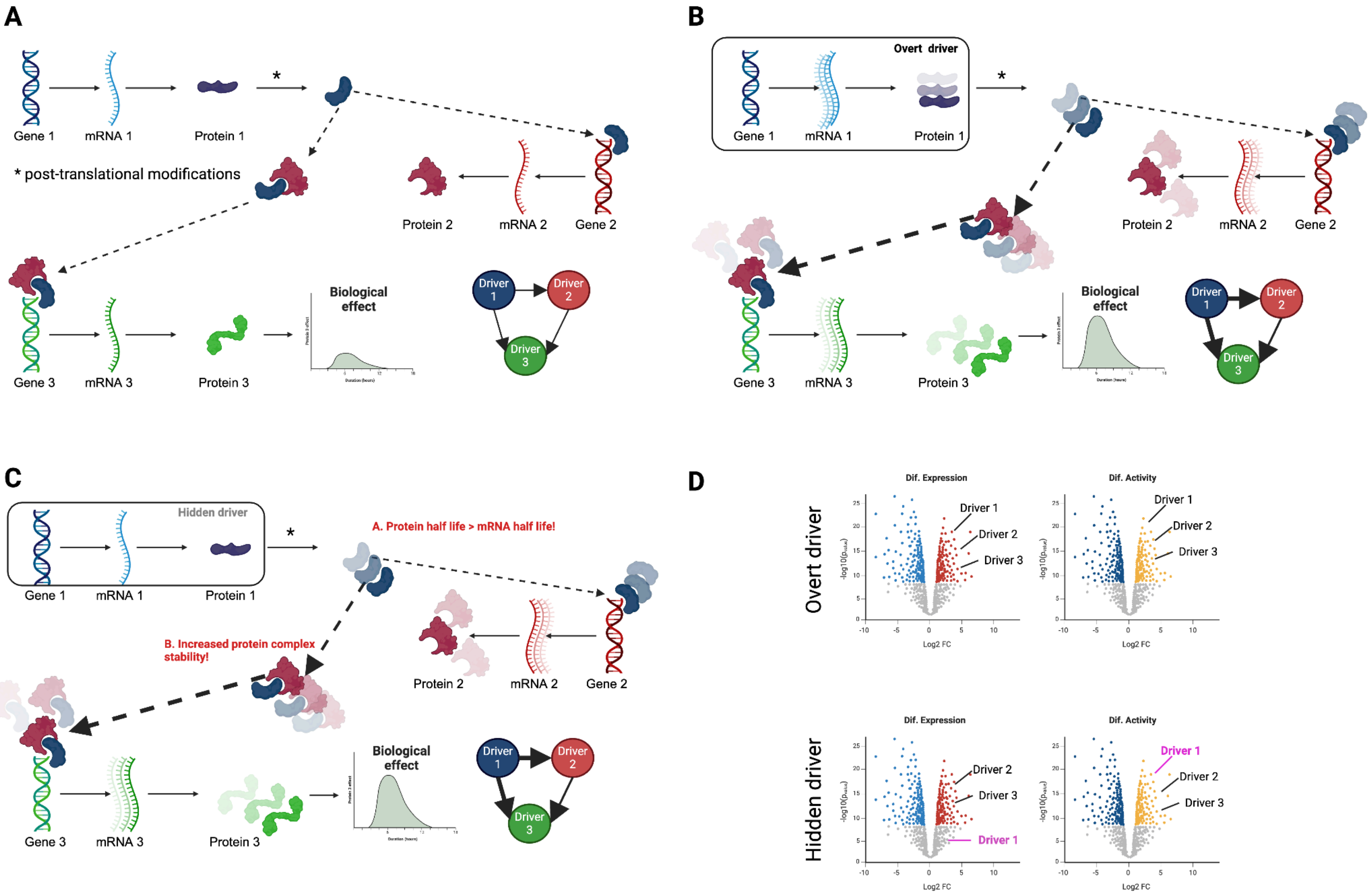

Figure SN1. The juxtaposition of activity and expression concepts in omic analysis. (A) Gene 1 activates the expression of Gene 2 as a transcription factor and then along with the product of Gene 2 regulates the expression of Gene 3 which confers the biological effect. **(B)** Amplification of expression of Gene 1 confers a cascade effect leading to the overexpression of Gene 2 and Gene 3, ultimately triggering a higher biological effect; in this case, Gene 1 is an *overt driver*, completely detectable from typical differential expression analysis **(C)** In certain cases, Gene 1 can exhibit no change in its expression but due to non-linear dynamics and post-translation modifications like increased protein half-life and increased protein-complex stability, the far-reaching biological effects through increased Gene 2 and 3 is retained; in this case, Gene 1 operates as a hidden driver, remaining obscure from typical differential expression analysis.

intracellular compartmentalisation of signalling events. Typical differential expression analysis can overlook crucial drivers of disease phenotypes. Therefore, tools like NetBID2 and scMINER are invaluable for addressing this gap in computational analysis. Figure SN1 provides a schematic representation of this juxtaposition between expression and activity.

These tools facilitate the context-specific reverse engineering of Gene or Protein Regulatory Networks (GRN/PRNs), formulating mechanistic hypotheses about signalling motifs and potential molecular targets in disease settings.
